# Identification and characterization of two *Isatis indigotica O*-methyltransferases methylating *C*-glycosylflavonoids

**DOI:** 10.1093/hr/uhac140

**Published:** 2022-06-23

**Authors:** Yuping Tan, Jian Yang, Yinyin Jiang, Shufu Sun, Xiaoyan Wei, Ruishan Wang, Junling Bu, Dayong Li, Liping Kang, Tong Chen, Juan Guo, Guanghong Cui, Jinfu Tang, Luqi Huang

**Affiliations:** State Key Laboratory of Dao-di Herbs, National Resource Center for Chinese Materia Medica, China Academy of Chinese Medical Sciences, Beijing 100700, China; School of Traditional Chinese Medicine, Shenyang Pharmaceutical University, Shenyang 117004, China; State Key Laboratory of Dao-di Herbs, National Resource Center for Chinese Materia Medica, China Academy of Chinese Medical Sciences, Beijing 100700, China; State Key Laboratory of Dao-di Herbs, National Resource Center for Chinese Materia Medica, China Academy of Chinese Medical Sciences, Beijing 100700, China; State Key Laboratory of Dao-di Herbs, National Resource Center for Chinese Materia Medica, China Academy of Chinese Medical Sciences, Beijing 100700, China; School of Pharmacy, Anhui University of Chinese Medicine, Hefei 230012, China; State Key Laboratory of Dao-di Herbs, National Resource Center for Chinese Materia Medica, China Academy of Chinese Medical Sciences, Beijing 100700, China; College of Chinese Medicinal Materials, Jilin Agricultural University, Changchun 130118, China; State Key Laboratory of Dao-di Herbs, National Resource Center for Chinese Materia Medica, China Academy of Chinese Medical Sciences, Beijing 100700, China; State Key Laboratory of Dao-di Herbs, National Resource Center for Chinese Materia Medica, China Academy of Chinese Medical Sciences, Beijing 100700, China; National Engineering Research Center for Vegetables, Beijing Vegetable Research Center, Beijing Academy of Agriculture and Forestry Science, Beijing 100097, China; State Key Laboratory of Dao-di Herbs, National Resource Center for Chinese Materia Medica, China Academy of Chinese Medical Sciences, Beijing 100700, China; State Key Laboratory of Dao-di Herbs, National Resource Center for Chinese Materia Medica, China Academy of Chinese Medical Sciences, Beijing 100700, China; State Key Laboratory of Dao-di Herbs, National Resource Center for Chinese Materia Medica, China Academy of Chinese Medical Sciences, Beijing 100700, China; State Key Laboratory of Dao-di Herbs, National Resource Center for Chinese Materia Medica, China Academy of Chinese Medical Sciences, Beijing 100700, China; State Key Laboratory of Dao-di Herbs, National Resource Center for Chinese Materia Medica, China Academy of Chinese Medical Sciences, Beijing 100700, China; State Key Laboratory of Dao-di Herbs, National Resource Center for Chinese Materia Medica, China Academy of Chinese Medical Sciences, Beijing 100700, China; School of Traditional Chinese Medicine, Shenyang Pharmaceutical University, Shenyang 117004, China

## Abstract

*Isatis indigotica* accumulates several active substances, including *C*-glycosylflavonoids, which have important pharmacological activities and health benefits. However, enzymes catalyzing the methylation step of *C*-glycosylflavonoids in *I. indigotica* remain unknown. In this study, three *O*-methyltransferases (OMTs) were identified from *I. indigotica* that have the capacity for *O*-methylation of the *C*-glycosylflavonoid isoorientin. The Type II OMTs IiOMT1 and IiOMT2 efficiently catalyze isoorientin to form isoscoparin, and decorate one of the aromatic vicinal hydroxyl groups on flavones and methylate the C6, C8, and 3′-hydroxyl positions to form oroxylin A, wogonin, and chrysoeriol, respectively. However, the Type I OMT IiOMT3 exhibited broader substrate promiscuity and methylated the C7 and 3′-hydroxyl positions of flavonoids. Further site-directed mutagenesis studies demonstrated that five amino acids of IiOMT1/IiOMT2 (D121/D100, D173/D149, A174/A150R, N200/N176, and D248/D233) were critical residues for their catalytic activity. Additionally, only transient overexpression of Type II OMTs *IiOMT1* and *IiOMT2* in *Nicotiana benthamiana* significantly increased isoscoparin accumulation, indicating that the Type II OMTs *IiOMT1* and *IiOMT2* could catalyze the methylation step of *C*-glycosylflavonoid, isoorientin at the 3′-hydroxyl position. This study provides insights into the biosynthesis of methylated *C*-glycosylflavonoids, and IiOMTs could be promising catalysts in the synthesis of bioactive compounds.

## Introduction

Flavonoids, with a wide variety of bioactivities, have significant potential in the treatment of microbial infection, inflammation, and cancer, but their application and development are limited by their instability, low cell absorption, and low bioavailability. *O*-Methylation is a common and important modification of flavonoids in plants that changes their physiological properties, including stability and solubility [3–5], facilitating absorption [6], and higher bioavailability [7]. It has been reported that polymethoxylated 4′-deoxyflavones, such as skullcapflavone I and tenaxin I, showed higher ability to induce human cancer cell apoptosis than baicalein [8]. Methoxylated flavonoids are limited in their applications as therapeutic drugs because of low yields in plants, the tedious nature of their chemical synthesis, and low purity levels. Enzymatic methylation exhibits high efficiency, stereo- and regioselectivity to create methoxy bonds directly and sustainably. Therefore, it is a promising method for synthesizing methoxy flavonoids [9].

The *O*-methylation of flavonoids in plants is strictly controlled by *O*-methyltransferases (OMTs), and the reported plant flavonoid *O*-methyltransferases were classified into two types [10]. Type I OMTs are larger enzymes, ranging in size from 38 to 43 kDa, and utilize substrates with a high degree of structural diversity. These enzymes include isoliquiritigenin OMT1 and isoflavonoid OMT from *Medicago sativa* [11] (ChOMT and IOMT), isoflavonoid 4′-OMT from *Glycine max* [12] (GmIOMT1), flavonoids 7-OH from *Perilla frutescens* [13] (PfOMT3), flavonoids 3′-OMT from *Oryza sativa* [14] (ROMT-9) and *Arabidopsis thaliana* [15] (AtOMT1), flavonols 3′/5′-OMT from *Catharanthus roseus* [16] (CrOMT2), tricetin 3′,4′,5′-OMT from *Triticum aestivum* [17] (TaOMT2), flavones 3,3′,5,7-OMT from *Citrus depressa* [18] (CdFOMT5), flavonoids 3′,5′,7-OMT from *Citrus reticulata* [19], and ObFOMTs from *Ocimum basilicum*, methylating hydroxyl groups at specific flavonoid positions [20]. These enzymes catalyze the *O*-methylation of flavonoids without any metal cations. Cation-dependent OMTs (Type II OMTs) are smaller enzymes that range in size from 23 to 29 kDa and require divalent cations for their activity. Most Type II OMTs methylate caffeoyl coenzyme A (CCoAOMTs) during lignin biosynthesis [21, 22]. PFOMT from *Mesembryanthemum crystallinum* is the first Mg^2+^-dependent *O*-methyltransferase that can catalyze flavonols, aside from its traditional substrate caffeoyl-CoA [23]. These enzymes were later discovered in other species. *At4g26220* from *A. thaliana* methylates 4′-OH or 3′-OH of flavanones and dihydroflavonols [24]. PaF6OMT from *Plagiochasma appendiculatum* methylates 6-OH of baicalein and scutellarein to form oroxylin A and hispidulin, respectively [25]. PFOMTs from *Scutellaria baicalensis* methylate norwogonin, baicalein, and luteolin to form wogonin, oroxylin A, and chrysoeriol, respectively [8]. Additionally, several Type II OMTs have been discovered to methylate anthocyanin in plants. AOMT and FAOMT methylate 3′,5′-OH at the B-ring of anthocyanin in *Vitis vinifera* [26, 27]. In *Cyclamen*, CkmOMT2 catalyzes 3′ or 3′,5′ *O*-methylation at the B ring of glycosylated anthocyanidins involved in flower coloration [28]. PpAOMT2 methylates glycosylated anthocyanidins at the 3′ position in *Prunus persica* [29], while AnthOMT from *Solanum lycopersicum* has a strong affinity for glycosylated anthocyanidins [30]. However, little is known about the methyltransferases involved in the biosynthesis of *C*-glycosylflavonoids in plants.

*Isatis indigotica* is an essential medicinal herb in the Cruciferae, cultivated worldwide for its therapeutic properties [31, 32]. Its dried leaves are known as ‘Da Qing Ye’ and are used to treat encephalitis B, mumps, influenza, and leptospirosis [33–35]. Previous studies have found that dried leaves from *I. indigotica* accumulated a wide variety of *C*-glycosylflavonoids, such as isovitexin, isoorientin, isoscoparin, vicenin-2, and their derivatives. These compounds have made significant contributions to pharmacological activities [34, 35]; in particular, methylation of the *C*-glycosylflavonoid isoorientin drastically inhibited the inactivation of important adipogenic transcription factors in adipogenesis and provides a potential dietary supplement [36, 37]. However, the biosynthesis of *C*-glycosylflavonoids in *I. indigotica* remains unknown at the molecular level, and enzymes capable of catalyzing the methylation of the biosynthesis of *C*-glycosylflavonoids have not been identified. In this study, we analyzed transcriptome data on *I. indigotica* and found that two *I. indigotica* IiOMTs could methylate the 3′-hydroxyl position of the *C*-glycosylflavonoid isoorientin.

## Results

### Cloning and functional expression of putative methyltransferases involved in isoscoparin biosynthesis in *I. indigotica*

A BLASTP search of the *I. indigotica* transcriptome was performed using *AtOMT1* (AAM64800.1) from *A. thaliana* as a query, while three unigenes from two types of *OMT* were obtained as putative methyltransferases. The full-length cDNAs of the *IiOMT*s were amplified with the cDNA of *I. indigotica* leaves as a template, and were designated as *IiOMT1*, *IiOMT2*, and *IiOMT3*, respectively. The open reading frames (ORF) of the three *IiOMT* genes were 744, 699, and 1095 bp and were predicted to encode proteins of 257, 232, and 364 amino acids, respectively. The predicted amino acid sequences of the candidate *IiOMT* cDNAs corresponded to a 28.93-kDa protein, a 26.24-kDa protein, and a 39.74-kDa protein; all three proteins contained a conserved domain identified as methyltransferase (https://www.ncbi.nlm.nih.gov/Structure/cdd/wrpsb.cgi).

### Transcription expression of three candidate *IiOMTs* in different organs of *I. indigotica*

**Figure 1:**
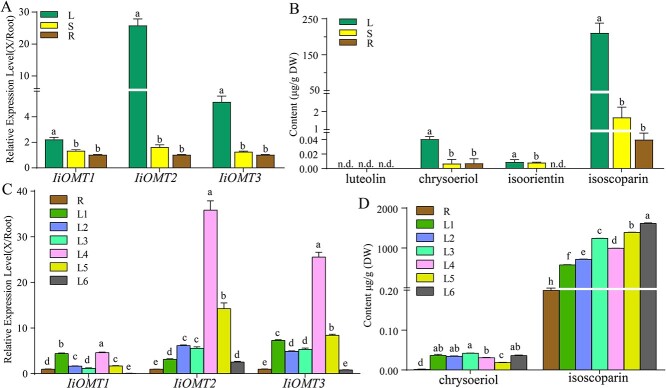
Expression levels of candidate *IiOMTs* in different organs and the accumulation of chrysoeriol and isoscoparin in *I. indigotica*. (A) Relative expression levels of *IiOMT1*, *IiOMT2*, and *IiOMT3* in different organs of *I. indigotica*. (B) Quantities of luteolin, chryseoriol, isoorientin, and isoscoparin from different organs in *I. indigotica.* (C) Expression patterns of *IiOMT*s in developing *I. indigotica* leaves. (D) Quantities of chrysoeriol and isoscoparin in developing *I. indigotica* leaves. ‘X/Root’ means that the expression of *IiOMT* in root was used as a reference experiment. R, roots; S, stems; L, leaves. L1 to L6 represent leaves at different maturation stages; L1 and L6 represent the youngest and early senescence leaves respectively. All data represent the mean ± standard deviation of three biological replicates. Different letters above the error bars indicate significant differences (*P* < .05) according to Tukey’s test.

We further investigated the expression patterns of the three candidate *IiOMTs* in different organs of *I. indigotica*. Three *IiOMT* genes were constitutively expressed in leaves, stems, and roots of *I. indigotica*, with the strongest expression in the leaves (Fig. 1A). Meanwhile, *IiOMT2* was expressed at a level that was 25.24-fold higher in leaves than in roots. To explore the relationship between the *IiOMTs* and correlated methylflavonoids, the amounts of methylflavonoids, chryseoriol, and isoscoparin were estimated in each organ. Large amounts of isoscoparin were found in *I. indigotica* leaves, while lower levels were found in the stems and roots (Fig. 1B). Chryseoriol also mainly accumulated in the leaves, although at lower levels than isoscoparin. The accumulation patterns of these methylflavonoids were consistent with those of *IiOMT* transcripts.

Due to the high expression levels of the three *IiOMTs* in leaves, we further investigated their expression patterns in leaves at different maturation stages (named L1 to L6, L1 and L6 representing the youngest and early senescence leaf, respectively). The expression level of *IiOMT1* was relatively higher in the youngest leaves and L4 leaves. Expression levels of *IiOMT2* and *IiOMT3* exhibited an overall increasing tendency in young leaves; levels were highest in L4 leaves and then decreased in senescence leaves (Fig. 1C). In leaves at different maturation stages, the amount of chryseoriol showed no significant change in young leaves (L1 to L4), but down-accumulated in more mature leaves (L5) and then returned to the basal level (Fig. 1D). Overall, the accumulation of isoscoparin showed an increasing tendency, with high expression levels of *IiOMTs* in early-stage leaves (L1 to L3), slightly decreased expression in L4 leaves, and high accumulation in early senescence with lower expression levels of *IiOMT*s (Fig. 1D). These results suggested that the expression patterns of these *IiOMTs* and the accumulation of chrysoeriol and isoscoparin were not strictly consistent during the leaf’s maturation.

### Phylogenetic analysis of the three candidate IiOMTs

**Figure 2:**
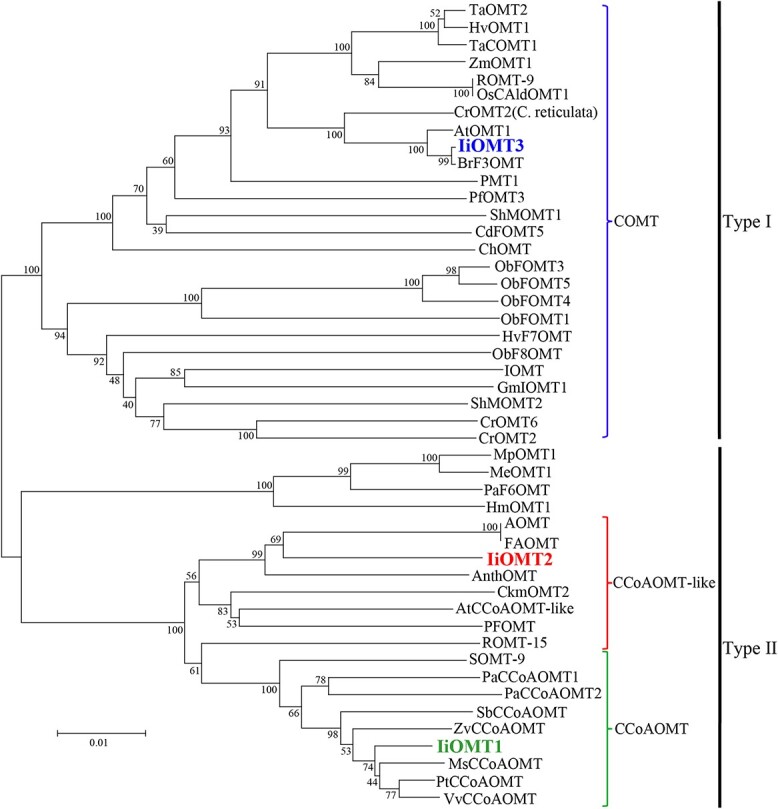
Phylogenetic tree of candidate IiOMTs and previously characterized plant methyltransferases. The neighbor-joining method was used to construct this tree with 1000 bootstrap replicates by MEGA7. The GenBank accession numbers of OMT proteins in this tree are in Supplementary Data Table S2

For further insight into potential functions of IiOMTs, we aligned the three protein sequences with similar and previously characterized plant OMTs that have flavonoid or phenylpropanoid activity and generated a phylogenetic tree. Although IiOMT1 and IiOMT2 shared a high amino acid sequence identity of 54.0% and belonged to Type II OMTs, IiOMT1 and IiOMT2 were placed in the caffeoyl CoA-OMT (CCoAOMT) subgroup and the CCoAOMT-like subgroup, respectively (Fig. 2). Additionally, IiOMT1 presented 90, 89, and 88% identity at the amino acid level with VvCCoAOMT (CAA90969.1) from *V. vinifera*, PtCCoAOMT (AAA80651.1) from *Populus tremuloides*, and MsCCoAOMT (AAC28973.1) from *M. sativa*, respectively. Interestingly, IiOMT1 also shared high homology with SOMT-9 from *G. max* (soybean). SOMT-9 methylates substrates with ortho-hydroxyl groups at a 3′-OH position including phenylpropanoids, as well as coumarins and flavonoids [14]. IiOMT2 was 93% similar to the CCoAOMT-like protein of *A. thaliana* (AAB96879.1), which methylates flavonoids, coumarin, and aromatic esters. Furthermore, the OMTs in this subgroup, including AnthOMT from *S. lycopersicum* [31], AOMT and FAOMT from *V. vinifera*, methylate anthocyanins and flavonoid glycosides at the 3′-OH and 5′-OH positions [26, 27]. IiOMT3 was grouped into a clade of Type I OMTs showing flavonoid activity and was 93.0% similar at the amino acid level to AtOMT1 from *A. thaliana*, which can methylate flavonoids at the 3′-OH position [15]. Thus, the evidence indicated that the three IiOMTs may be involved in the biosynthesis of methylflavonoids in *I. indigotica*.

**Figure 3:**
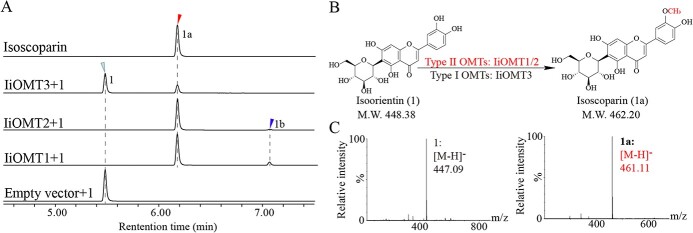
Functional characterization of candidate IiOMT proteins. (A) UPLC chromatograms of the reactions of IiOMTs with SAM and isoorientin as substrates. (B) A proposed biosynthetic pathway for isoscoparin in *I. indigotica*. (C) MS spectra of compounds 1 and 1a in negative mode. The maximum UV absorption peaks of the reactions are 350 nm.

### Heterogeneous expression of *IiOMT*s in *E. coli* and enzymatic activity assays *in vitro*

To identify the functional role of each of the three OMTs *in vitro*, their coding sequences were expressed in *E. coli* Rosetta (DE3). These three IiOMT proteins with a hexahistidine (His) tag and a SUMO tag at the *N*-terminus were highly expressed, and then purified using Ni-affinity chromatography. The molecular weights of the IiOMT fusion proteins were consistent with their expected values (Supplementary Data Fig. S1B).

IiOMT activity was tested *in vitro* with the potential substrate, isoorientin, in the presence of SAM. As shown in Fig. 3, IiOMT1 and IiOMT2 converted isoorientin (compound 1) into compounds 1a and 1b, while IiOMT3 only converted isoorientin (1) into 1a. The mass spectrum of 1a exhibited an [M-H]^−^ ion at *m*/*z* 461, which had a retention time and an MS pattern identical to the authentic sample of isoscoparin (Fig. 3B). To clarify the reaction product (1a) of isoorientin, NMR experiments were carried out. The ^1^H-NMR spectrum of the reaction product (1a) of isoorientin showed a signal at 3.95 ppm; meanwhile, it showed a signal at 56.7 ppm at the ^13^C NMR spectrum (Supplementary Data Fig. S2). Therefore, a methoxy group was produced by IiOMTs acting on the 3′-OH position of isoorientin. The mass spectrum of 1b showed an [M-H]^−^ ion at *m*/*z* 475, indicating that 1b was a dimethyl product (Supplementary Data Fig. S3). Luteolin is believed to be another important precursor in isoscoparin biosynthesis in *I. indigotica*; thus, enzymatic activity assays were also conducted using luteolin as the substrate. In the presence of SAM, three IiOMTs efficiently catalyzed luteolin into a single methylated product that had a retention time and an MS pattern identical to the authentic sample of chrysoeriol (Supplementary Data Fig. S5A and B). The results suggested that these three IiOMTs could be involved in flavonoid biosynthesis in *I. indigotica*, while IiOMT1 and IiOMT2 likely contribute to isoscoparin biosynthesis.

**Figure 4:**
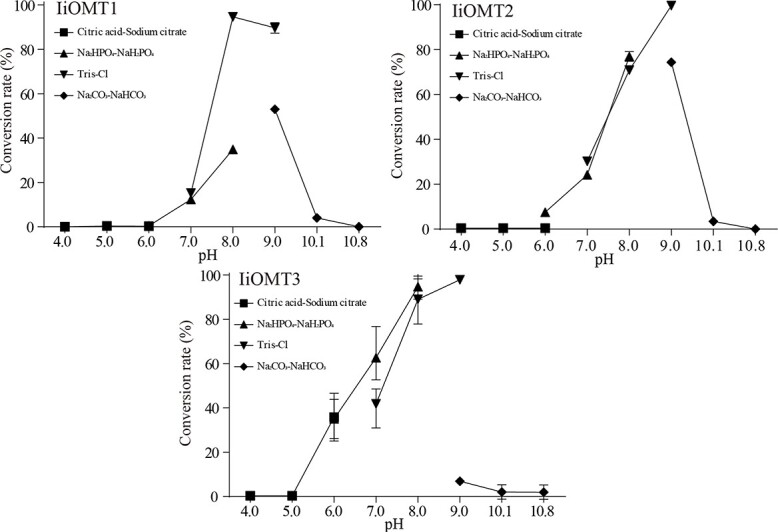
Effect of pH on the activities of purified IiOMT proteins. To verify pH preference for reactions, reactions were examined using isoorientin as a substrate, as described in the Materials and methods section. All data represent the mean ± standard deviation of three biological replicates. The maximum activity levels of IiOMT were assumed to be 100%.

### Biochemical characterization of the recombinant IiOMT proteins

**Figure 5:**
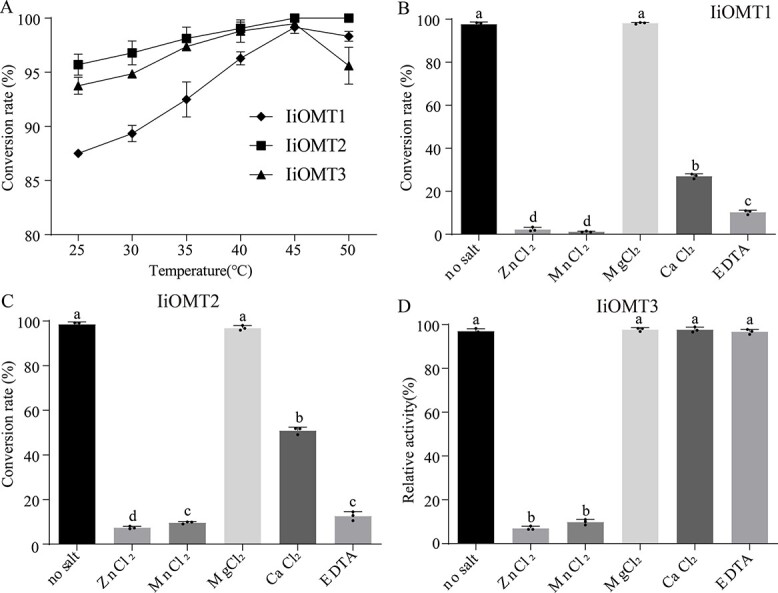
Properties of purified proteins. Effect of temperature (A) and divalent cations (B–D) on IiOMT enzyme activities for isoorientin. All data represent the mean ± standard deviation of three biological replicates. The maximum activity levels of IiOMT were assumed to be 100%. Different letters above the error bars indicate significant differences (*P* < .05) according to Tukey’s test.

The enzymatic properties of IiOMTs were examined. Different pH ranges, temperatures, and reaction times were assayed to optimize the IiOMT reaction conditions. The optimal pH for IiOMT1 activity was determined to be 8.0, with low activity below pH 7.0 and 9.0, with Tris-HCl as buffer. The most suitable buffer for both IiOMT2 and IiOMT3 was Tris–HCl (pH 9.0) (Fig. 4). Under the different tested temperatures, all IiOMTs exhibited the highest catalytic activity at 45°C (Fig. 5A). The effect of divalent cations on IiOMT activities was also tested. The activities of both IiOMT1 and IiOMT2 were unaffected by Mg^2+^ (Fig. 5B and C). Though the activity of IiOMTs was affected to some degree in the presence of various divalent cations, the activities of both IiOMT1 and IiOMT2 were extremely low in the presence of EDTA, while the activity of IiOMT3 was not affected by EDTA (Fig. 5B–D). This indicates that IiOMT1 and IiOMT2, which harbor divalent cation binding sites, were cation-dependent methyltransferases, while IiOMT3 was a cation-independent OMT.

The kinetic properties of IiOMTs were determined within the linear range of the enzymatic reaction. Each purified recombinant protein was subjected to a reaction at 45°C and an optimal pH in Tris–HCl buffer with various concentrations of luteolin and isoorientin. As shown in Table 1, the apparent *K*_m_ value of IiOMT3 for luteolin (41.49 μM) was the highest; however, the *K*_cat_/*K*_m_ value of IiOMT3 for luteolin (366.62 μM^−1^ s^−1^) was the lowest. The apparent *K*_m_ and *K*_cat_/*K*_m_ values of IiOMT1 and IiOMT2 for luteolin were 113.5 μM and 3645.33 μM^−1^ s^−1^, and 77.03 μM and 1225.23 μM^−1^ s^−1^, respectively. Additionally, both IiOMT1 and IiOMT2 showed comparable affinity to isoorientin, whose values toward isoorientin were 75.56 and 62.08 μM for *K*_m_, and 4035.65 and 1289.91 μM^−1^ s^−1^ for *K*_cat_/*K*_m_, respectively. In contrast, the catalytic efficiency of IiOMT3 for isoorientin (778.3 μM for *K*_m_, 0.52 × 10^−2^ μM^−1^ s^−1^ for *K*_cat_/*K*_m_) was relatively weaker. Overall, IiOMT1 was the more efficient enzyme for the methylation of luteolin and isoorientin compared with IiOMT2.

**Table 1:** Kinetic parameters of recombinant IiOMTs.

	Luteolin			Isoorientin		
	IiOMT1	IiOMT2	IiOMT3	IiOMT1	IiOMT2	IiOMT3
*K*_m_ (μmol/L)	113.5 ± 10.41	77.03 ± 12.60	41.49 ± 7.67	75.56 ± 8.21	62.08 ± 16.11	778.3 ± 16.90
*V*_max_ (nmo/min.mg)	600.40 ± 12.57	146.50 ± 9.16	17.50 ± 1.11	442.50 ± 12.41	124.30 ± 10.88	0.49 ± 0.03
*K*_cat_ (s^−1^)	4.13 × 10^−1^	9.43 × 10^−2^	1.52 × 10^−2^	3.04 × 10^−1^	8.01 × 10^−2^	4 × 10^−4^
*K*_cat_/*K*_m_ (M^−1^ s^−1^)	3645.33	1225.23	366.62	4035.65	1289.91	0.52 × 10^−2^

Results are mean ± standard deviation of three independent experiments.

### Catalytic regioselectivity of IiOMTs

**Figure 6:**
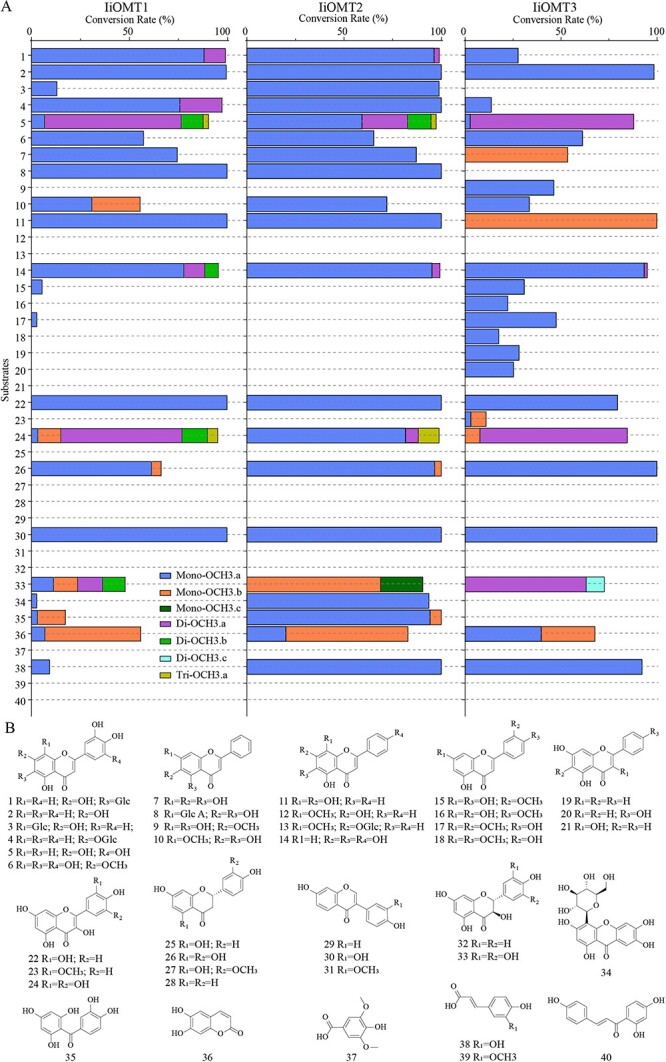
Substrate promiscuity of three IiOMTs for *O*-methylation. (A) Conversion rate of methylated products for substrates 1–40. (B) Structures of substrates 1–40.

To explore the catalytic regioselectivity of IiOMTs, a total of 40 compounds with varied structures were used for methylation reactions containing only one hydroxyl group on a specific site, an ortho-hydroxyl group or a hydroxymethyl group on the A-ring or B-ring (Fig. 6). Liquid chromatography–mass spectrometry (UPLC/Q-TOF-MS) analysis indicated that three IiOMTs catalyzed different structural types of flavonoids with an ortho-hydroxyl group on the B-ring, including flavone (compound 2, luteolin), flavonol (compound 22, quercetin), flavanonol (compound 26, eriodictyol), and isoflavone (compound 30, 3′-hydroxydaizein). The main products for compounds 22, 26, and 30 were identified as 3′-methylflavonoids by comparing the retention time and the MS pattern with the commercial standards (Supplementary Data Figs S5C and D, S6A and B, and S6C and D).

To identify additional functions of the IiOMTs, we also tested other flavonoid substrates. Both IiOMT1 and IiOMT2 could also catalyze the *O*-methylation of flavone glycosides (compounds 3, 4, and 8), and the conversion rates of IiOMT2 exceeded 98.9% for flavone glycosides. With baicalein as acceptor, IiOMT1 and IiOMT2 could convert baicalein (compound 7) into 6-methylflavone (oroxylin A) with SAM as a methyl donor, whereas IiOMT3 could produce 7-methylflavone (negletein) (Supplementary Data Fig. S4C and D). With norwogoninas the acceptor, both IiOMT1 and IiOMT2 could methylate norwogonin (compound 11) at 8-OH to form wogonin in the presence of S-adenosylmethionine, whereas IiOMT3 could produce another methylflavone (Supplementary Data Fig. S5A and S5B). It is noteworthy that IiOMT2 showed robust catalytic capabilities toward flavonoids or flavone glycosides with an ortho-hydroxyl group (up to 100% conversion). However, IiOMT2 could not catalyze the methylation of a single hydroxyl or an ortho-hydroxymethyl of flavonoids, including flavones (compounds 9, 12, and 15–20), flavonols (21 and 23), flavanonols (27 and 28), isoflavones (29 and 31), and 2-hydroxyflavaone (32). This indicated that the ortho-hydroxy group is necessary for substrates to be acceptable to IiOMT2. In contrast, IiOMT1 and IiOMT3 could also use chrysoeriol (compound 15) and diosmetin (compound 17) as acceptor molecules, although the efficiency levels were not high. In addition, IiOMT3 could catalyze the methylation of a single hydroxyl of flavones, including chrysin (compound 19) and apigenin (compound 20). On the other hand, IiOMT3 not only used flavones with a hydroxymethyl group chrysoeriol (compound 15), velutin (17), and isorhamnetin (23), which have 3′-OCH3 and 4′-OH at the B-ring, as acceptor molecules, but could also methylate diosmetin (compound 16) and pillion (18), which have 3′-OH and 4-OCH_3_ at the B-ring. Interestingly, IiOMTs could methylate esculetin (36) and caffeic acid (38), which possess aromatic vininal dihydroxy groups but could not accept syringic acid (37) and ferulic acid (39) as substrates.

Altogether, the results indicated that IiOMTs could efficiently methylate the 3′-OH position of vicinal hydroxyl group flavonoids at the B-ring, but IiOMT3 showed weaker or loss of catalytic capabilities toward substrates with introduced sugar molecules. Additionally, IiOMT1 and IiOMT2 methylated the C6- and 8-hydroxyl positions to form oroxylin A and wogonin, respectively, while IiOMT3 exhibited broader substrate promiscuity and methylated the C7-hydroxyl positions of flavonoids.

**Figure 7:**
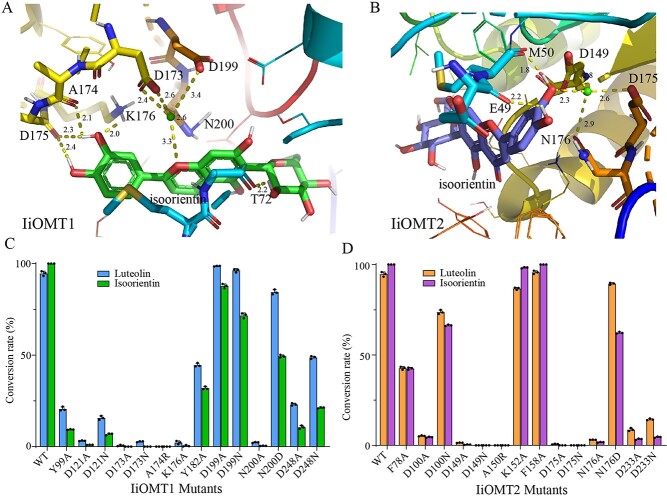
Molecular modeling and site-directed mutagenesis revealed the key residues of IiOMT1 and IiOMT2. Homology-based models of the active sites of IiOMT1 (A) and IiOMT2 (B) interacted with isoorientin. (C, D) Site-directed mutagenesis to identify the essential residues of IiOMT1 (C) and IiOMT2 (D).

**Figure 8:**
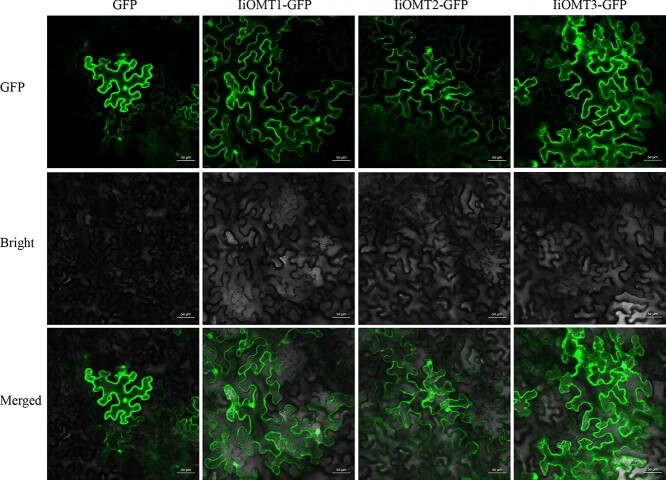
Subcellular localization of IiOMTs tagged at the C-terminus with GFP in *N. benthamiana*. GFP, GFP channel; Bright, light microscopy image; Merged, merged image of the GFP and Bright channels. Scale bars are 50 μm.

### Site-directed mutagenesis of IiOMT1 and IiOMT2 identified critical residues for catalytic activity

The different catalytic activity of IiOMT1 and IiOMT2 led us to investigate their protein structure and key residues determining catalytic patterns. Homology models for IiOMT1 based on *Medicago* CCoAOMT (PDB ID: 1sui) and IiOMT2 based on *Sorghum* CCoAOMT (PDB ID: 5kva) were created after optimal templates were identified. In both structures, the key residues of divalent metal ions were almost identical, but residues that interacted with the acceptors differed in each structure (Fig. 7A and B, Supplementary Data Fig. S8). As shown in Fig. 7A, in IiOMT1 residue Asp175 forms two hydrogen bonds with the oxygen at 4′-OH and the oxygen at 3′-OH of isoorientin. Residue Ala174 forms a hydrogen bond with the oxygen at 3′-OH of isoorientin. Additionally, the side-chain carboxylic moieties of Asp173 and Asn200 and the side chain Oδ of Asp173 and Asp199 coordinated the Mg^2+^ ion in a bidentate or monodentate manner, and the O1 moiety of isoorientin coordinated the Mg^2+^ ion. However, in IiOMT2, the side-chain carboxylic moieties of Asn176 and the side-chain Oδ of Asp149 and Asp175 coordinated the Mg^2+^ ion in a monodentate manner and the oxygen at 3′-OH of isoorientin coordinated the Mg^2+^ ion (Fig. 7B). We analyzed the structure docking results and sequence alignment of IiOMTs to other plants’ OMTs and found that the residues in the coordination of the Mg^2+^ ion were conserved in both IiOMTs, as Asp173/Asp199/Asn200 in IiOMT1 and Asp149/Asp175/Asn176 in IiOMT2 (Supplementary Data Fig. S8).

Site-directed mutagenesis and enzyme assays demonstrated that five amino acid mutations of IiOMT1/IiOMT2 (D121/D100, D173/D149, A174/A150, N200/N176, and D248/D233) decreased and almost abolished the methylation activity to two optimal substrates (Fig. 7C and D). Further protein sequence alignment and phylogenetic analyses indicated that these five amino acids were highly conserved in Type II OMTs, while N200/N176 was only found in Type II OMTs (Supplementary Data Fig. S8). The D121A, D173A, A174R, and N200A mutants of IiOMT1 decreased to <5% activity compared with the wild type. The residues (Y99/Y182 in IiOMT1) were conserved in the crystal structures of MsCCoAOMT and SbCCoAOMT, leading us to mutate these residues in IiOMT1 (Supplementary Data Fig. S8). The mutation of Y99, Y182, and D248 to Ala reduced activity to 23.02, 48.04, and 24.15% toward luteolin, respectively, and 9.58, 32.91, and 11.60% toward isoorientin, respectively, compared with the wild type of IiOMT1. There is only a small difference between residues Asp and Asn in the side chain, therefore catalytic properties of mutants D121N, D173N, N200D, and D248N of IiOMT1 were further investigated. Compared with the wild type, the D121N, D173N, and D248N mutants of IiOMT1 impaired the enzyme activity to varying degrees, or even abolished the enzyme activity, suggesting that the hydroxyl moieties of the side chains of residues D121, D173, and D248 play an important role in the substrate combination. However, the N200D mutant of IiOMT1 retained high activity toward the test substrates, which may be due to the binding of the carboxylic moiety rather than the amine moiety of the side chain of Asn200 to the substrates. Similar to IiOMT1, the D100A, D149A, D149N, A150R, N176A, D233A, and D233N mutants of IiOMT2 retained <10% activity toward the substrates. Residues Y99 and Y182 in IiOMT1 were replaced by Phe in IiOMT2 (F78 and F158) and mutation of F78 to Ala reduced activity to 45.29 and 42.93% for luteolin and isoorientin, respectively, but mutation of F158 to Ala catalyzed methylation almost at the original level. Residue K176 in IiOMT1 corresponds to K152 in IiOMT2. The IiOMT1 mutant (K176A) almost abolished the methylation activity, whereas the IiOMT2 mutant (K152A) almost retained original activity toward the test substrates. On the contrary, mutation of residue D199 of IiOMT1 to Ala nearly retained original activity toward the test substrates, while the mutation of the residue in the corresponding location of IiOMT2 (D175) to Ala or Asn severely impaired enzyme activity. Overall, five amino acids between IiOMT1 and IiOMT2 (D121/D100, D173/D149, A174/A150R, N200/N176, and D248/D233) demonstrated that they are highly conserved in Type II OMTs and play important roles in the *O*-methylation. Additionally, the K176A mutation that affected enzyme activity was specific to IiOMT1 while the D175A that affected enzyme activity was specific to IiOMT2.

### Subcellular localization analysis of IiOMTs

To examine the localization of IiOMTs, the recombinant IiOMT fused with GFP in the C-terminal was transiently expressed in *N. benthamiana* leaves. As shown in Fig. 8, the fluorescent signals of three IiOMT fusion GFP proteins were primarily distributed throughout the cytoplasm, as well as in the nucleus. This indicated that the catalytic activity of these IiOMTs was present in the cytoplasm and that the cytoplasm could be the subcellular site for the biosynthesis of isoscoparin.

### Transient overexpression of *IiOMT*s in *N. benthamiana*

**Figure 9:**
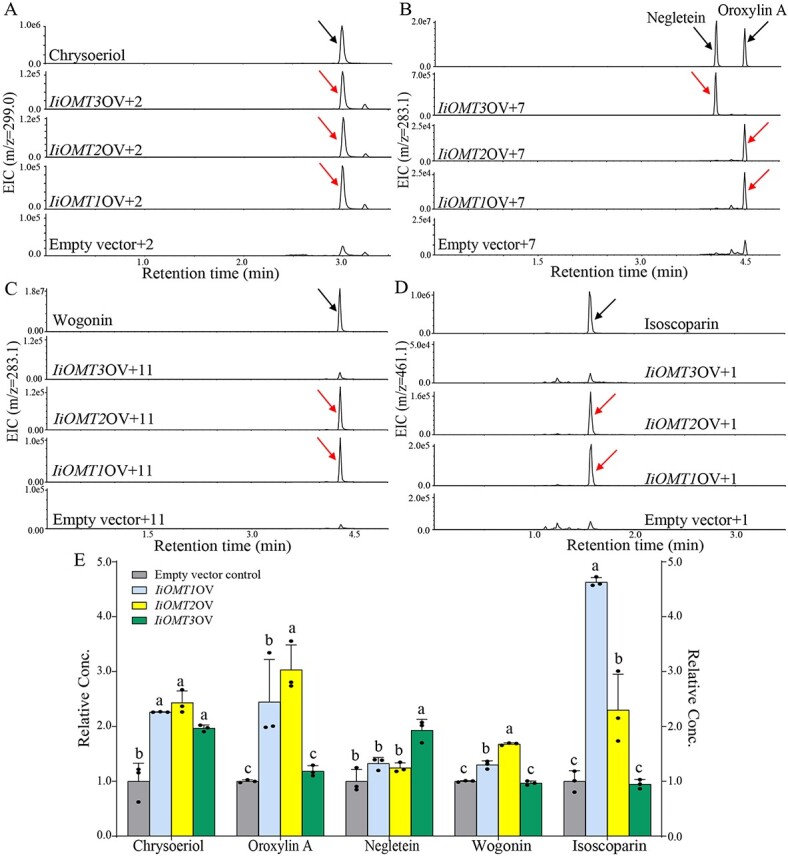
Transient overexpression analysis of *IiOMTs* in *N. benthamiana*. (A–D) LC–MS extracted ion chromatograms (EIC) of *N. benthamiana* leaves with *IiOMT*s or empty vector adding luteolin (compound 2), baicalein (7), norwogonin (11), and isoorientin (1), respectively. (E) Effect of transient overexpression of *IiOMT* genes on the contents of chrysoeriol, oroxylin A, negletein, wogonin, and isoscoparin. Data are mean ± standard deviation of three biological replicates. Different letters above the error bars indicate significant differences (*P* < .05) according to Tukey’s test.

**Figure 10:**
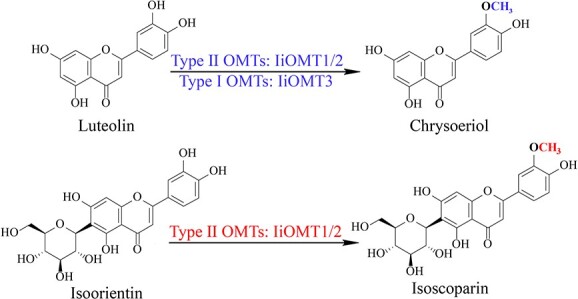
Catalytic activity of *I. indigotica* IiOMTs in isoscoparin biosynthesis.

To further investigate the catalytic activity of *IiOMT*s, we overexpressed *IiOMTs* under the control of the 35S promoter in *N. benthamiana* leaves*.* Leaves with an infiltrated suspension of *A. tumefaciens* with 35S empty vector were used as a control. The fluorescent signals of three IiOMT fusion GFP proteins were found to be distributed throughout the cytoplasm, suggesting that three *IiOMT*s were expressed in *N. benthamiana* (Fig. 8). We found no differences in the two major products between 35S:*IiOMT*-infiltrated leaves and 35S empty vector-infiltrated leaves, and even oroxylin A, negletein and wogonin were not detected in *N. benthamiana* leaves, which could be due to the extremely low accumulation of the precursors in *N. benthamiana* (Supplementary Data Fig. S11). In contrast, there were significant differences in the accumulation of methylflavonoids between 35S:*IiOMT*-infiltrated leaves and 35S empty vector-infiltrated leaves when they were infiltrated with the substrates, 2 days after infiltration of *A. tumefaciens* (Fig. 9). Compared with the control, the accumulation of chrysoeriol was 2.26-, 2.43-, and 1.96-times higher in leaves infiltrated with 35S:*IiOMT* than in leaves infiltrated with 35S empty vector (Fig. 9A and E). In addition, baicalein (compound 7) or norwogonin (compound 11) was infiltrated in *N. benthamiana*. As expected, the expression of IiOMT1 and IiOMT2 in *N. benthamiana* resulted in a significant accumulation of oroxylin (6-methylflavone) and wogonin (8-methylflavone), while the expression of IiOMT3 yielded negletein (7-methylflavone) in high abundance in *N. benthamiana* leaves (Fig. 9B, C, and E). However, only overexpression of Type II OMTs, *IiOMT1* and *IiOMT2*, increased the accumulation of isoscoparin in *N. benthamiana* leaves, which was 4.63 and 2.50 times higher than that in 35S empty vector-treated leaves, respectively (Fig. 9D and E). These results demonstrated that two types of OMT enzymes methylated flavonoid aglycones, but only Type II OMTs, IiOMT1 and IiOMT2, could methylate the *C*-glycosylflavonoid isoorientin at the 3′-hydroxyl position (Fig. 10)*.*

## Discussion

Several plant *O*-methyltransferases have been discovered, but no OMTs have been identified that can catalyze methylation in the biosynthesis of *C*-glycosylflavonoids, such as isoscoparin. Several studies have demonstrated that *I. indigotica* leaves accumulate plentiful *C*-glycosylflavonoids such as isoorientin, isovitexin, isoscoparin, and their derivatives, indicating the importance of the molecular mechanism of isoscoparin biosynthesis in *I. indigotica* [37, 38]. In this study, we found that two types of OMTs with different catalytic features could be involved in the biosynthesis of isoscoparin in *I. indigotica*. Both Type I OMT, IiOMT3, and Type II OMTs, IiOMT1 and IiOMT2, efficiently catalyzed *O*-methylation at the 3′-OH position of luteolin; however, only Type II OMTs, IiOMT1 and IiOMT2, showed high catalytic capabilities towards isoorientin to form isoscoparin.

The process of biosynthesis and accumulation of *C*-glycosylflavonoids in *I. indigotica* is mainly carried out in the leaves [33]. In this study, we investigated the relationship between expression patterns of these *IiOMTs* and the accumulation of chrysoeriol and isoscoparin in leaves at different maturation stages. Overall, during the leaf’s maturation, the expression levels of *IiOMT2* and *IiOMT3* maintained an increasing tendency, then decreased to a low level in early senescence leaves, while a high level of *IiOMT1* was observed in the youngest and mature leaves and remained at a low level in leaves at other maturation stages. In leaves at different maturation stages, the amount of chryseoriol remained relatively higher in young stages (L1 to L4), but decreased in more mature stages (L5) and then returned to the basal level. This observation may suggest that chryseoriol was an intermediate product during the biosynthesis of flavonoids and preferred to transform into downstream compounds, and thus the amount of chryseoriol returned to low-level accumulation in senescence leaves. In contrast, the amount of isoscoparin showed an increasing tendency with high expression levels of *IiOMT*s in early-stage leaves L1 to L3; accumulation remained high in early senescence, with lower expression levels of *IiOMT*s. This result indicated that isoscoparin was a main storage form of methylated *C*-glycosylflavonoids *in planta*. This notion was also supported by the observation that the amount of isoscoparin was still higher in early senescence than in mature leaves. Our finding also supported the idea that non-active glycosylated forms of compounds are stored to avoid autotoxicity in plants, which may release toxic aglycones in response to the attack [39, 40].

The known Type I OMTs have been confirmed to directly methylate flavonoid aglycones in many species, including *G. max*, *O. sativa*, and *C. reticulata*; however, C-glycosyltransferases in plants typically use non-methylated flavonoid aglycones as acceptors, such as daidzein, apigenin, luteolin, or 2-hydroxynaringenin [41]. Genetic studies of anthocyanin methylation have confirmed that anthocyanin methylation occurs after glycosylation rather than directly on the aglycones [28, 29]. In this study, we demonstrated that Type II OMTs, IiOMT1 and IiOMT2, can efficiently catalyze *O*-methylation reactions after *C*-glycosylation during the biosynthesis of isoscoparin in *I. indigotica*. The *K*_m_ values for IiOMT1 and IiOMT2 indicated that they prefer the substrate isoorientin, not luteolin. IiOMT1 and IiOMT2 catalyzing the *O*-methylation of isoorientin was consistent with the accumulation of large amounts of isoscoparin, but not chrysoeriol or isoorientin, in *I. indigotica* leaves (Fig. 1). Further, transient overexpression of *IiOMT1* and *IiOMT2* in *N. benthamiana* could increase the accumulation of isoscoparin compared with the control. Additionally, the overexpression of *IiOMT1* and *IiOMT2* in *N. benthamiana* accumulated much higher levels of isoscoparin but not chrysoeriol compared with the control*.* This indicated that Type II OMTs, IiOMT1 and IiOMT2, were capable of efficiently producing the *C*-glycosylflavonoid isoscoparin. However, further *IiOMT1* and *IiOMT2* knockout transgenic lines in vivo were still needed *in vivo* were still needed to investigate their roles in the biosynthesis of methylated *C*-glycosylflavonoids *in planta*.

Although IiOMT1 shared high sequence identity with IiOMT2 (>50%), they were grouped into different subgroups in the Type II OMTs. Their substrate specificity and regioselectivity were distinctly different. IiOMT2 showed strict substrate stereoselectivity, only accepted glycosylflavonoids or phenylpropanoids with aromatic vicinal hydroxyl groups as substrates *in vitro*, and showed high catalytic capabilities (up to 100% conversion). However, the enzyme activity of IiOMT1 was detectable with flavones containing vicinal hydroxyl groups and hydroxymethyl groups. Sequence alignments of different plant Type II OMTs, including IiOMT1 and IiOMT2, demonstrated that the amino acid residues identified as putatively important for SAM binding and substrate binding were not the same (Supplementary Data Fig. S8). To test whether the amino acids located in conserved domains of IiOMT1 and IiOMT2 contribute to their distinct catalytic patterns, molecular docking and site-directed mutagenesis were performed. Although the five amino acids of IiOMT1/IiOMT2 (D121/D100, D173/D149, A174/A150R, N200/N176, and D248/D233) indicate that those highly conserved in Type II OMTs play an important role in *O*-methylation, our molecular docking indicated that the Mg^2+^ coordination geometry differed for IiOMT1 and IiOMT2. These interactions, which are shared between Mg^2+^ and IiOMT1, can help stabilize the substrate for the reaction [21, 42]. The side-chain oxygens of D173, D199, and N200 are involved in the chelation of the Mg^2+^ ion. However, D199 within 3.4 Å may have a weak interaction with the Mg^2+^ ion, meaning the replacement of conserved D199 with Ala did not abolish activity toward the substrates (Fig. 7A and C). Compared with D175, A174 and K176 were closer to the 3′-hydroxyl group of isoorientin, which could easily initiate the *O*-methylation. As such, these two IiOMT1 mutants (A174R and K176A) do not methylate at a detectable level, and the D175 mutant retained its methylation activities. In IiOMT2, the side-chain oxygens of D149, D175, and N176 were involved in the chelation of the metal ion, which could mediate the deprotonation of the isoorientin 3′-hydroxyl group and contribute to facile transmethylation [43]. We also mutated the highly conserved residues between IiOMT1 and IiOMT2, which may be important for catalysis, substrate binding, or substrate specificity in Type II OMTs [21], However, the mutation of these residues in both IiOMT1 and IiOMT2 retained most of its original activity toward the test substrates (Supplementary Data Fig. S10). This indicated that more data are needed to identify the catalytic mechanism of OMTs. Altogether, this analysis revealed the catalytic mechanism behind how IiOMT1 and IiOMT2 catalyze the *O*-methylation of isoorientin.

Flavonoid methylation is typically attributed to Type I OMTs. These enzymes often mediate the methylation of flavonols (such as CrOMT2 from *C. roseus* [16] and ShMOMT2 from *Solanum habrochaites* [44]), flavones (such as ROMT-9 from *O. sativa* [14]), or isoflavones (such as IOMT from *M. sativa* [11] and GmIOMT1 from *G. max* [12]). Though OMTs such as CrOMT2, ROMT-9, and IiOMT3 can catalyze the *O*-methylation of flavonols and flavones, they have different substrate catalytic properties. For example, ROMT-9 exhibits relatively broad substrate regiospecificity for flavonoids, with a preference for the 3′-hydroxy group, but it did not perform catalytic activity for the 7-hydroxy group of flavones compared with IiOMT3. The identification of IiOMT3 from *I. indigotica* based on the similarity to AtOMT1, which shared 93.0% amino acid sequence identity, suggested the structural conservation of flavonoid methyltransferases [15]. Although they are similar and form an independent phylogenetic clade based on the amino acid sequence, flavonoid methyltransferases show functional diversity. Compared with AtOMT1, IiOMT3 exhibits broader substrate promiscuity and regioselectivity: it methylated the 3′-hydroxy group of flavonoids such as luteolin, eriodictyol, and 3′-hydroxydaizein, catalyzed caffeic acid to form ferulic acid, and methylated the 7-OH positions of flavones.

In summary, we characterized two types of *O*-methyltransferases that could modify methylated *C*-glycosylflavonoids in *I. indigotica*. We determined that IiOMT1 could play a critical role in isoorientin modification and found that two types of *O*-methyltransferases catalyzed *O*-methylation at the 3′-OH position of luteolin in *I. indigotica*. Identifying these three IiOMTs provided novel insights into the biosynthesis of methylated *C*-glycosylflavonoids, which could be efficient biocatalysts capable of synthesizing methylated *C*-glycosylflavonoids and subsequently be used in medicinal treatments.

## Materials and methods

### Plant material, RNA extraction, and cDNA cloning

*Isatis indigotica* plants growing in the suburbs of Beijing were harvested in the autumn of 2017. Total RNA was extracted from different organs using Trizol reagent (Invitrogen, USA), and transcribed to cDNA with a PrimeScript™ RT Reagent Kit with gDNA Eraser. Full-length cDNAs of IiOMTs were amplified with specific primers using the cDNA of leaves as a template, and then cloned into pE-SUMO expression vectors (Supplementary Data Table S1).

### Expression patterns of putative *IiOMT*s in different organs

Total RNA was extracted from different organs, including roots, stems, and leaves, while cDNAs were synthesized with the total RNAs (2 μg each) using a PrimeScript™ RT Reagent Kit with gDNA Eraser. Gene expression levels were detected with TB Green Premix Ex Tap™ II (Tli RNaseH Plus) on a Roche LightCycler 480. The primers of *IiOMT*s for qRT–PCR are listed in Supplementary Data Table S1. The calibration curves of each pair of primers had a single peak, while quantification was performed using Roche 480 Analysis software.

### UPLC–MS/MS analysis

For ultraperformance liquid chromatography–tandem mass spectrometry (UPLC–MS/MS) analysis, roots, stems, and leaves of *I. indigotica* were freeze-dried and ground into powder. Fifty milligrams of each powdered sample was extracted with 1.5 mL methanol under sonication for 45 minutes and centrifuged (12 000 × g, 20 minutes at room temperature). The supernatants were filtered with 0.22-μm filters using a Waters C18 column (2.1 mm × 10 mm, 1.8 μm) with 0.1% formic acid–acetonitrile (A) and 0.1% acid–water (B) as mobile phases. The elution method was as follows: 5% A at the initial time; 25% A at 1 minute; 50% A at 4 minutes; 95% A at 4.5 minutes; 95% A at 6 minutes; 5% A at 7 minutes; 5% A at 9 minutes. Multiple monitoring methods were used to assess quantification, while the selected *m*/*z* transitions were 447.1 → 357.1 at 1.32 minutes for isoorientin, 461.1 → 341.1 at 1.52 minutes for isoscoparin, 285.0 → 133.0 at 2.33 minutes for luteolin, and 299.1 → 284.0 at 3.02 minutes for chrysoeriol.

### Molecular phylogenetic analysis of IiOMTs

The nucleotide sequences of IiOMTs were compared by blastn at NCBI (http://www.ncbi.nlm.nih.gov/), and the amino acid sequences were analyzed with Vector NTI Advance 11.5.3. A phylogenetic tree was constructed with plant OMT protein sequences using MEGA version 7.0 (Supplementary Data Table S2). The neighbor-joining method was used with 1000-replicate bootstrap support.

### Heterologous expression, purification, and catalytic parameters of recombinant IiOMT proteins

The full-length cDNAs of IiOMTs were amplified with specific primers and then inserted into the pE-SUMO expression vector at BamHI/SalI sites (Supplementary Data Table S1). After sequencing confirmation, the IiOMT-pE-SUMO construct was transformed into *Escherichia coli* Rosetta strain (DE3) for fusion protein expression. *E. coli* Rosetta 1 (DE3) cells harboring IiOMT-pE-SUMO were cultured at 37°C until the OD_600_ reached 0.6, and then induced with 0.5 mM isopropyl β-d-thiogalactoside (IPTG) at 16°C for 16 hours. The induced cells were harvested by centrifugation and then washed with sterile cold distilled water, centrifuged, resuspended with the buffer (50 mM Tris–HCl, 500 mM NaCl, pH 7.4), and sonicated on ice. Recombinant protein purification was carried out with Ni-NTA resin and concentrated using an Amicon-Ultra-0.5 Ultracel-10 k membrance. The purity of the His-tag-fused IiOMTs was examined with 10% SDS–PAGE glue, and the protein concentration was determined by a Bradford Protein Assay Kit with bovine serum as the standard (TransGen Biotech, Beijing, China).

To test the optimal pH of IiOMT enzyme activity, assays were performed in 50 mM citric acid–sodium citrate buffer (pH 4.0–6.0), 50 mM sodium phosphate buffer (pH 6.0–8.0), 50 mM Tris–HCl buffer (pH 7.0–9.0), and 50 mM sodium carbonate buffer (pH 9.0–10.8) with isoorientin as the substrate. To determine the optimal temperature for IiOMT activity, +the reactions were incubated at 25–55°C. The optimal quantity of pure protein was assessed in the 2–3000 μg range. MgCl_2_, CaCl_2_, ZnCl_2_, MnCl_2_, or EDTA was added individually at the final concentration of 10 mM to estimate the metals’ inhibition of enzyme activity. For kinetic studies, recombinant IiOMTs were incubated in a final volume of 100 μL with 480 μM *S*-adenosyl-L-methionine (SAM). Luteolin and isoorientin concentrations ranging between 5 and 2500 μM were used for determination of the Michaelis constant (*K*_m_). Data were adapted to the Michaelis–Menten equation, and a non-linear regression program was used to calculate the *V*_max_ and *K*_m_ values.

### Analysis of IiOMT reaction products

The enzymatic reactions (100 μL) included 480 μM SAM and 200 μM substrate. Pure proteins (5 μg IiOMT1, 5 μg IiOMT2, and 10 μg or 3 mg IiOMT3) were incubated at 45°C for 45 minutes. Double-volume methanol was then added to the assays to quench the reactions. The mixtures were clarified by centrifugation (12 000 g, 15 minutes at room temperature), and then the supernatant was filtered through a 0.22-μm nylon column and analyzed by liquid chromatography–mass spectrometry (UPLC/Q–TOF–MS). Separation was performed with a Waters ACQUITY UPLC HSS T3 (2.1 × 100 mm, particle size 1.8 μm). Mobile phases consisting of 0.1% (v/v) formic acid in acetonitrile (solvent A) and 0.1% (v/v) formic acid in water were used at a flow rate of 0.5 mL/minute and a UV wavelength from 200 to 400 nm. The elution gradient system was 10–21% A (0–3 minutes), 35–45% A (5–7 minutes), 55–65% A (9–12 minutes), 95% A (12.5–14 minutes), and 10% A (14–16 minutes).

### Scaled-up reactions and ^1^H and ^13^C data of methylated product of IiOMT2

The reaction system was amplified in a total volume of 30 mL IiOMT2 crude protein with 450 μM isoorientin and 480 μM SAM. The reaction mixture was extracted with two volumes of methanol, and dissolved with 2 mL methanol after the organic phase was pressurized and dried. Mobile phases consisting of acetonitrile and water (2:8, v/v) were used to separate the reaction product in a Shimadzu UFPLC system at a flow rate of 10 mL/minute and a UV wavelength of 350 nm using a C18 20 × 250 mm column. The separated product was determined using UPLC/Q–TOF–MS, ^1^H-nuclear magnetic resonance (NMR), and ^13^C-NMR. NMR spectra were recorded with a Bruker Ascend spectrometer and compared with the reported NMR spectrum for isoscoparin [38]: ^1^H-NMR (400 MHz, CD_3_OD) *δ* 7.50 (1H, d, *J* = 8.2 Hz, H-6′), 7.47 (1H, s, H-2′), 6.93 (1H, d, *J* = 8.2 Hz, H-5′), 6.64 (1H, s, H-3), 6.51 (1H, s, H-8), 4.90 (1H, d, *J* = 10.0 Hz, H-1′′), 3.95 (3H, s, 3′-OCH_3_), 3.87 (1H, brd, *J* = 11.9 Hz, H-6′a), 3.73 (1H, dd, *J* = 11.9, 5.0 Hz, H-6′b). ^13^C-NMR (100 MHz, CD_3_OD) δ 166.1 (C-2), 104.3 (C-3), 184.1 (C-4), 162.1 (C-5), 109.3 (C-6), 165.1 (C-7), 95.3 (C-8), 158.8 (C-9), 105.2 (C-10), 123.6 (C-1′), 121.8 (C-2′), 149.5 (C-3′), 152.2 (C-4′), 116.8 (C-5′), 110.6 (C-6′), 75.3 (C-1′′), 72.6 (C-2′′), 80.1 (C-3′′), 71.8 (C-4′′), 82.6 (C-5′′), 62.9 (C-6′′), 56.7 (3′-OCH_3_).

### Molecular docking

To build the 3D structures of IiOMT1 and IiOMT2 binding with isoorientin (1), we modeled their homology using SWISS-MODLE (https://swissmodel.expasy.org/) based on the *Medicago* CCoAOMT (PDB ID: 1sui) and *Sorghum* CCoAOMT (PDB ID: 5kva) crystal structure, respectively. Molecular docking between IiOMT and isoorientin was investigated using AutoDockTools 1.5.6.

### Site-directed mutagenesis of IiOMTs

The site-directed mutagenesis of IiOMTs at the residues was performed via PCR with IiOMT1-pE-SUMO or IiOMT2-pE-SUMO as a template. Corresponding primers are listed in Supplementary Data Table S1. PCR products were digested with a DMT enzyme and transformed into DMT *E. coli*. The sequences of the IiOMT-pE-SUMO mutants were confirmed via sequencing and transformed into Rosetta (DE3) *E. coli* for heterologous expression.

### Subcellular localization and transient expression of *IiOMT*s in *Nicotiana benthamiana*

The coding regions without IiOMT stop codons were cloned into the plant expression vector pCAMBIA1300-cGFP. Recombinant vectors carrying *IiOMT* and the empty vector pCAMBIA1300-cGFP were transferred into *Agrobacterium tumefaciens* strain GV3101. Transformants were cultured in LB medium containing 50 μg mL^−1^ kanamycin and 50 μg mL^−1^ rifampicin at 28°C, collected by centrifugation, and then resuspended in 10 mM MES buffer (10 mM MgCl_2_, 150 μM acetosyringone, pH 5.6) to a final OD_600_ of 1.0. After incubation for 2 hours at room temperature, the mixed *A. tumefaciens* was injected into 6-week-old *N. benthamiana* leaves. After 2 days, the green fluorescent protein (GFP) fluorescence of *N. benthamiana* leaves was captured using a Zeiss LSM 510 META confocal microscope.

For substrate feeding studies, 100 μM of luteolin or isoorientin in 0.1% DMSO in water was infiltrated into previously agroinfiltrated leaves. Leaves were harvested 1 day later and freeze-dried for metabolite analysis.

### Statistical analysis

Statistical analyses were performed using GraphPad Prism 8.0 software. Data are presented as mean ± standard error of the mean unless stated otherwise. *P*-values were calculated using Tukey’s test. Non-linear regression analysis was carried out to calculate kinetic parameters using the Michaelis–Menten model.

## Acknowledgements

This work was supported by the Scientific and Technological Innovation Project of the China Academy of Chinese Medical Sciences (CACMS Innovation Fund, C12021A04114), the National Natural Science Foundation of China (81974517, 81603241), the Fundamental Research Funds for the Central Public Welfare Research Institutes from the China Academy of Chinese Medical Sciences (ZZ11-115), and a key project at central government level: The Ability to Establish Sustainable use of Valuable Chinese Medicine Resources (2060302).

## Author contributions

Y.T.: conceptualization, methodology, and original draft preparation. J.Y.: methodology and NMR analysis. Y.J., S.S., X.W., and R.W.: methodology, software and visualization. L.K.: chemical analysis. T.C.: bioinformatics analysis. D.L., J.G., and G.C.: data analysis and reviewing. L.H.: funding acquisition, project administration, and resources. J.T.: investigation, validation, writing, reviewing, supervision, and editing.

## Data availability

All data supporting this research result can be obtained in this paper and in its supplementary materials published online.

## Conflict of interest

The authors declare no conflicts of interest.

## Supplementary data

Supplementary data is available at *Horticulture Research* online.

## Supplementary Material

supp_data_uhac140
