# Supplementary material for: Identification and characterization of two Isatis indigotica O-methyltransferases methylating C-glycosylflavonoids
Source: Hortic Res. 2022 Jun 23;9:uhac140. doi: 10.1093/hr/uhac140 (PMC9437721; doi:10.1093/hr/uhac140)
Supplement: supp_data_uhac140 [file supp_data_uhac140.zip › Supplementary data.pdf]

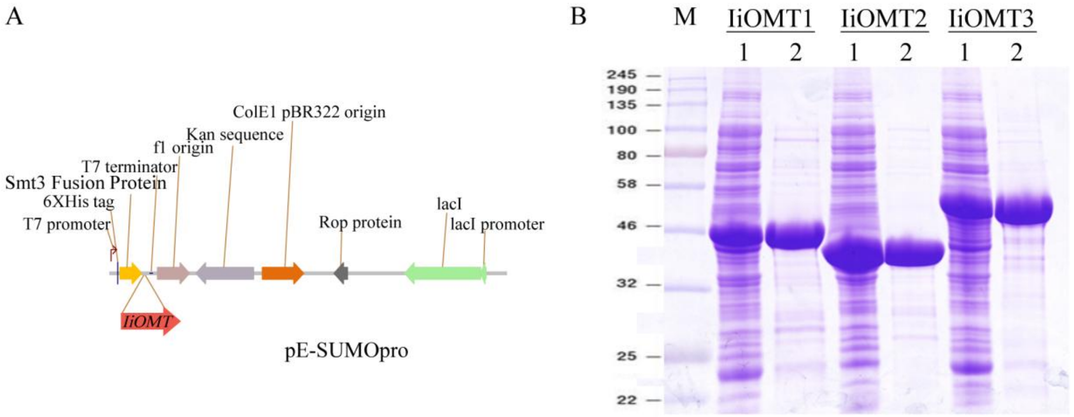

Figure S1. Expression of the *IiOMT* genes in *E. coli*.

(A) The full lenght ORF of *IiOMTs* were inserted into the pE-SUMOpro vector under control of the T7 promoter. (B) SDS-PAGE analysis of expression and purification of *IiOMT*-pE-SUMOpro. Lane M: molecular weight marker; Lane 1: The crude protein induced by IPTG; Lane 2: The purified protein.

A

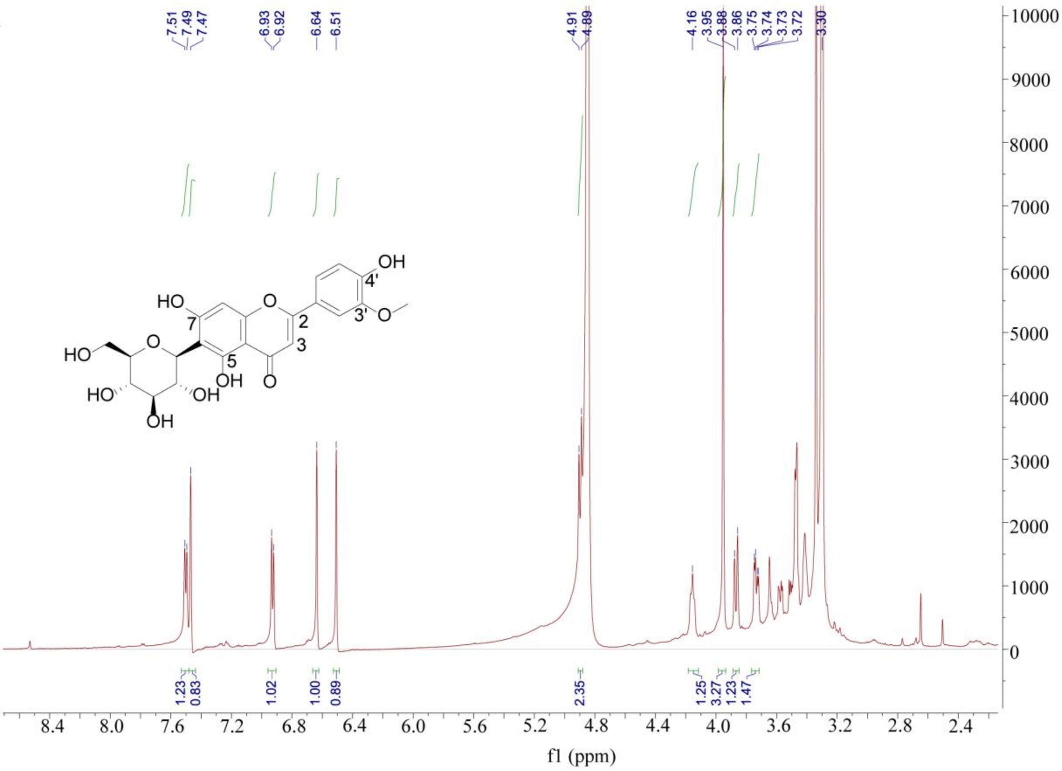
7.51 7.49 7.47 6.93 6.92 6.64 6.51 4.91 4.89 4.16 3.95 3.88 3.86 3.75 3.74 3.73 3.72 3.30
1.23 0.83 1.02 1.00 0.89 2.35 1.25 3.27 1.23 1.47
HO 7 5 OH O 2 3 3' 4' OH
HO HO OH OH
f1 (ppm)

B

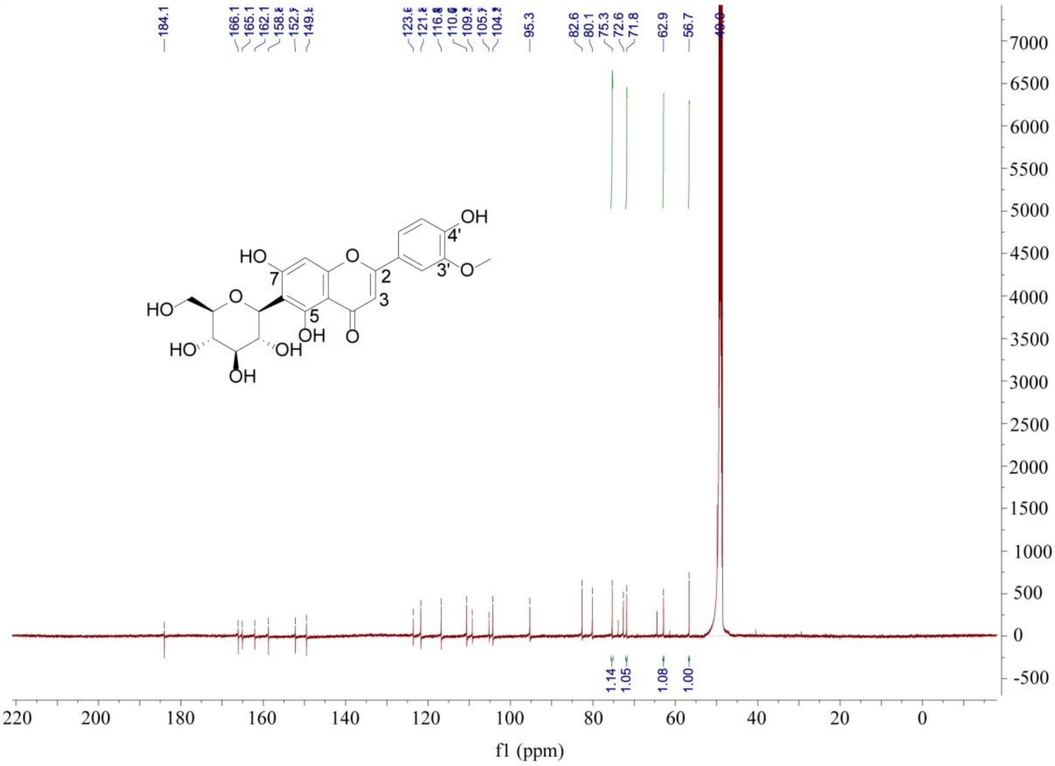
184.1 166.1 165.1 162.1 95.3 82.6 80.1 75.3 72.6 71.8 62.9 56.7
1.14 1.05 1.08 1.00
HO 7 5 OH O 2 3 3' 4' OH
HO HO OH OH
f1 (ppm)

Figure S2. The ^{1}H and ^{13}C NMR spectra of the product produced by IiOMT2 with isoorientin as a substrate.

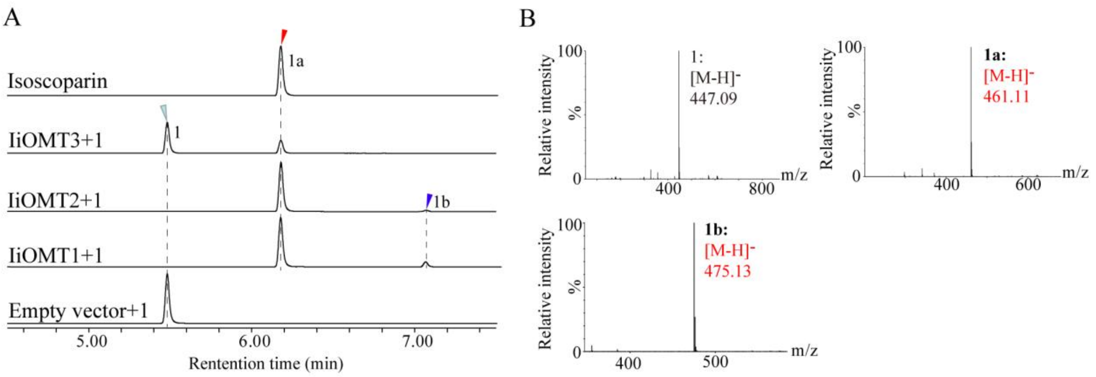

Figure S3. The IiOMTs-catalysed methylation of (1) isoorientin with SAM.
UPLC chromatograms of the reactions of IiOMTs with isoorientin (A). MS spectra of 1a and 1b in negative mode (B).

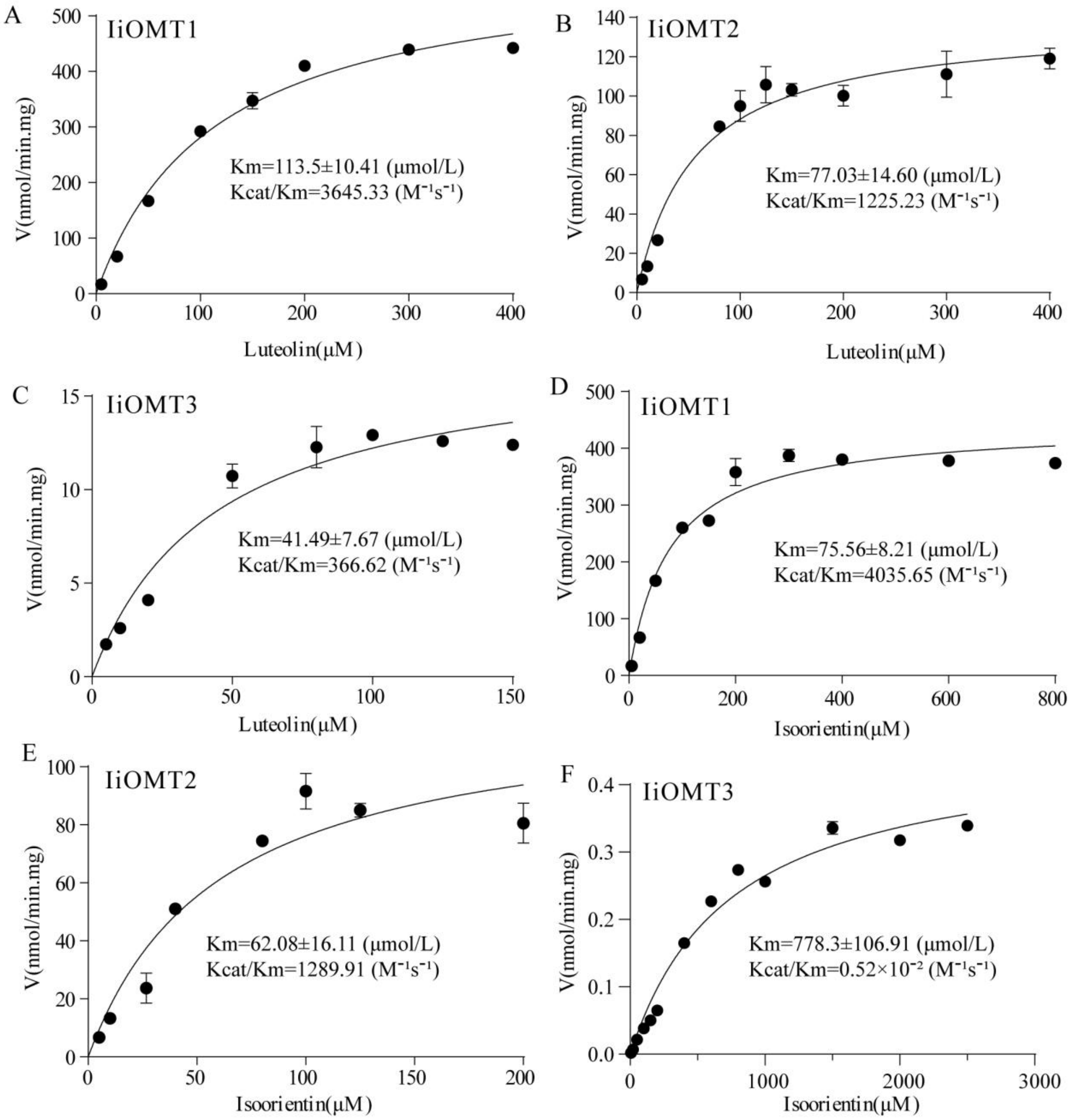

Figure S4. Kinetic parameters of recombinant IiOMTs proteins.

(A), (D) are showed kinetic parameters of IiOMT1 for luteolin and isoorientin, respectively. (B), (E) are showed kinetic parameters of IiOMT2 for luteolin and isoorientin, respectively. (C), (F) are showed kinetic parameters of IiOMT3 for luteolin and isoorientin, respectively. All data represent the mean±standard deviation (SD) of three biological replicates.

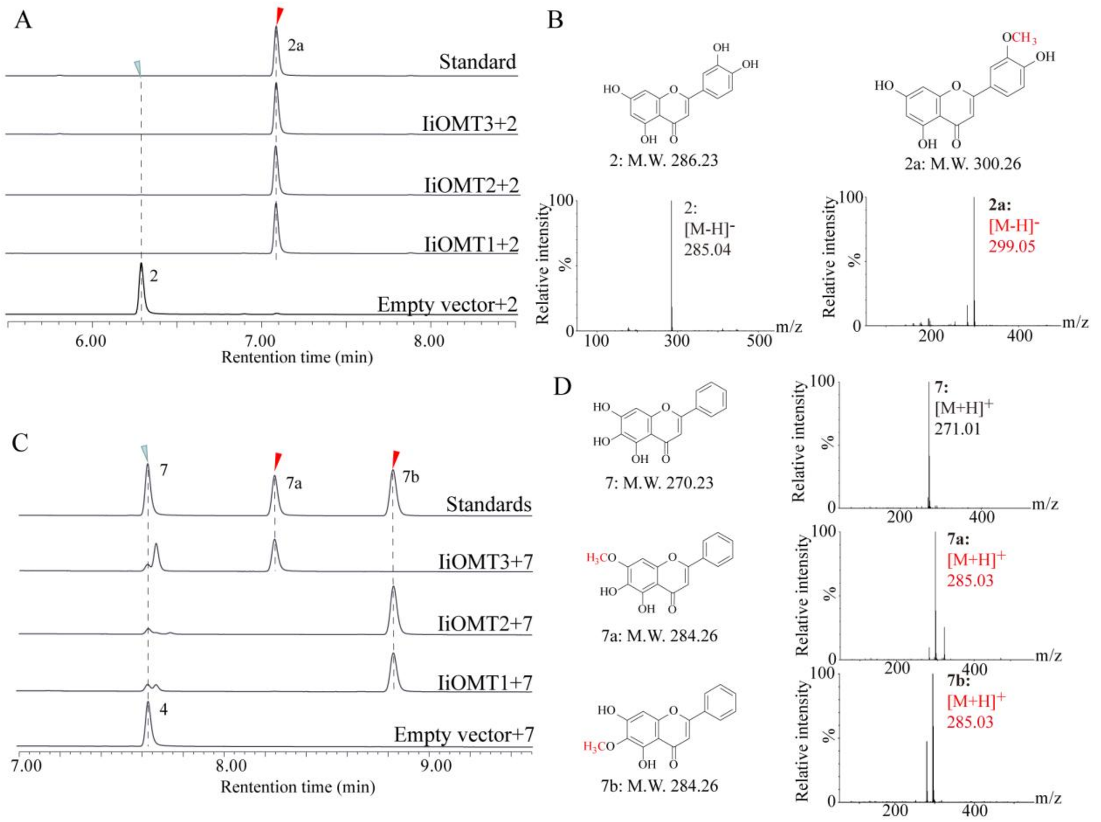

Figure S5. The IiOMTs-catalysed methylation of (2) luteolin and (7) baicalein with SAM, respectivey.

UPLC chromatograms of the reactions of IiOMTs with different receptors including luteolin (A) and baicalein (C). MS spectra of 2a in negative mode (B), and 7a and 7b in positive mode (D).

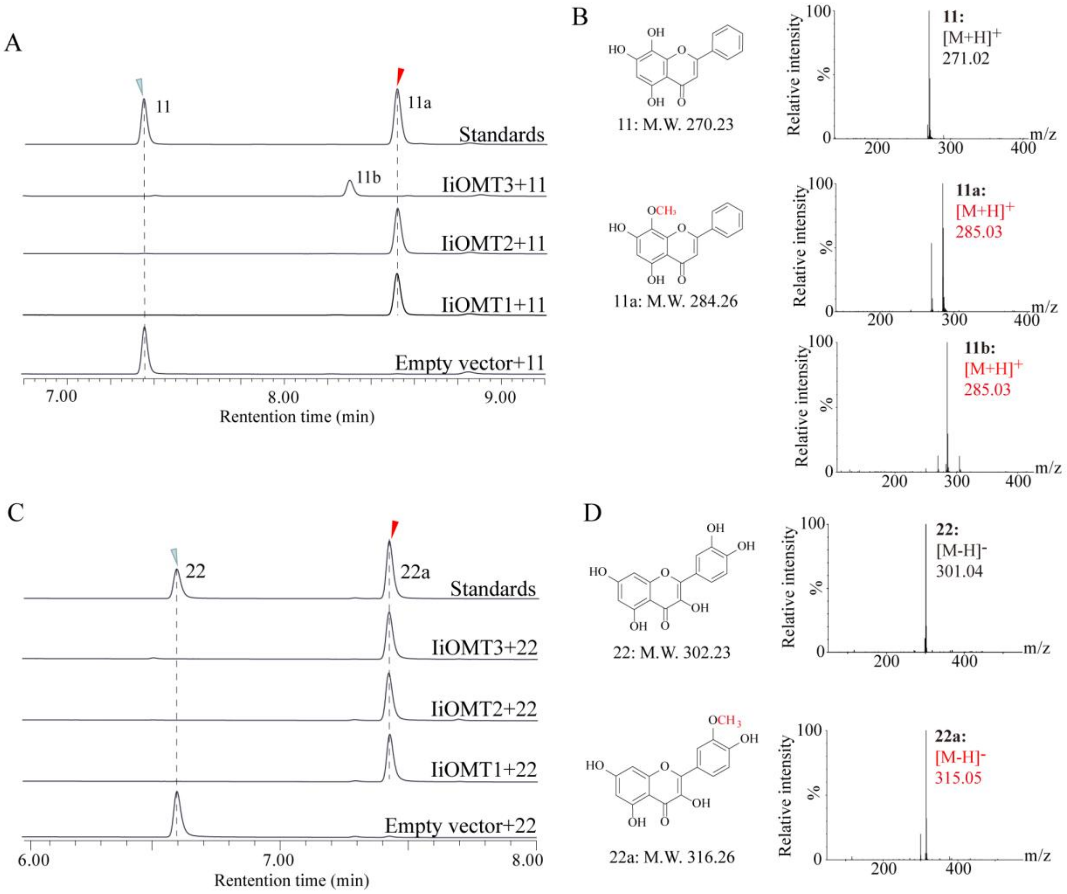

Figure S6. The IiOMTs-catalysed methylation of (11) norwogonin and (22) quercetin with SAM, respectivey.

UPLC chromatograms of the reactions of IiOMTs with different receptors including norwogonin (A) and quercetin (C). MS spectra of 11a and 11b in negative mode (B), and 22a in positive mode (D).

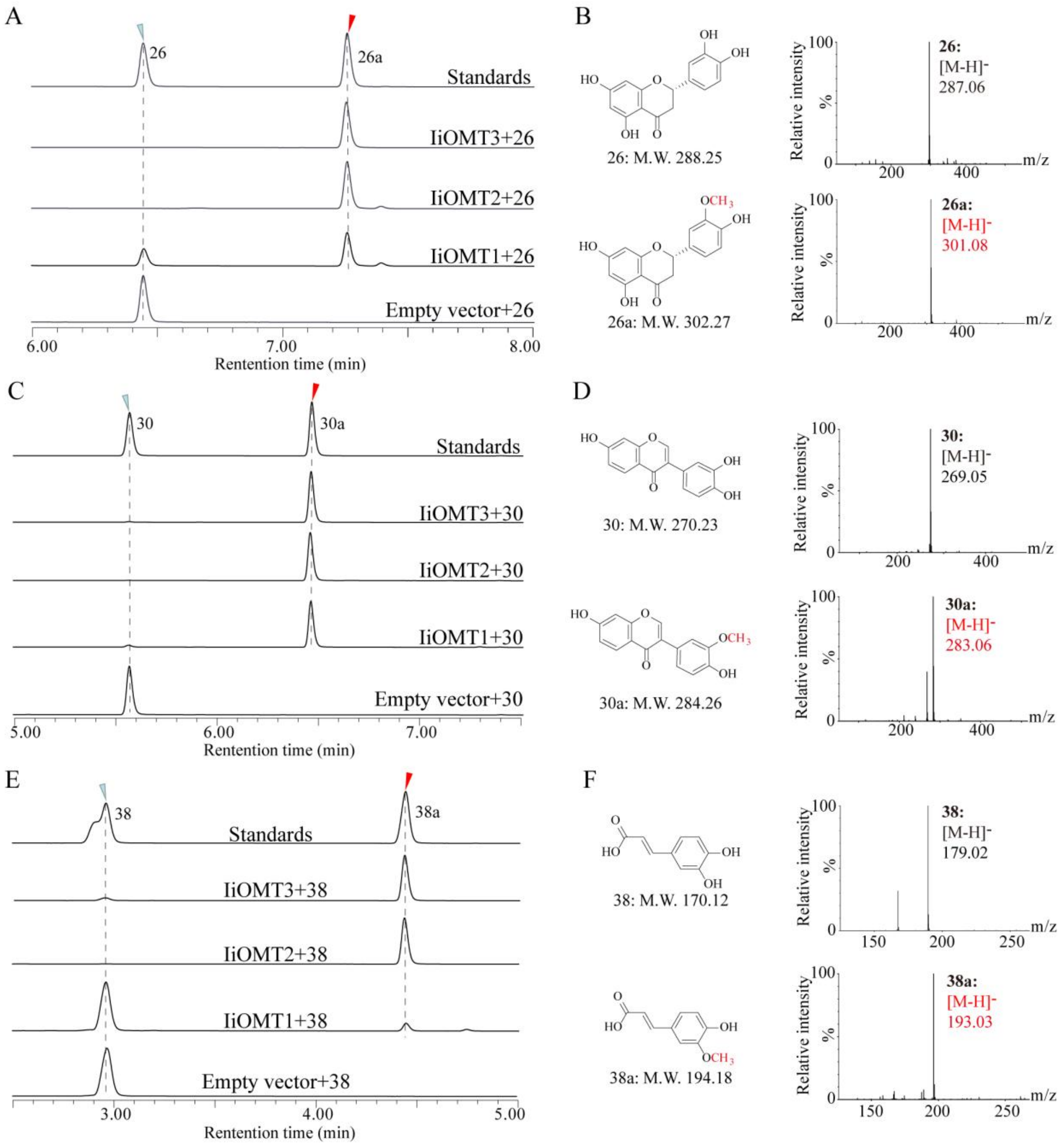

Figure S7. The IiOMTs-catalysed methylation of (26) eriodictyol, (30) 3'-hydroxydaidzein and (38) caffeic acid with SAM, respectivey.

UPLC chromatograms of the reactions of IiOMTs with different receptors including eriodictyol (A), 3'-hydroxydaizein (C) and caffeic acid (E). MS spectra of 26a (B), 30b (D) and 38a (F) in negative mode.

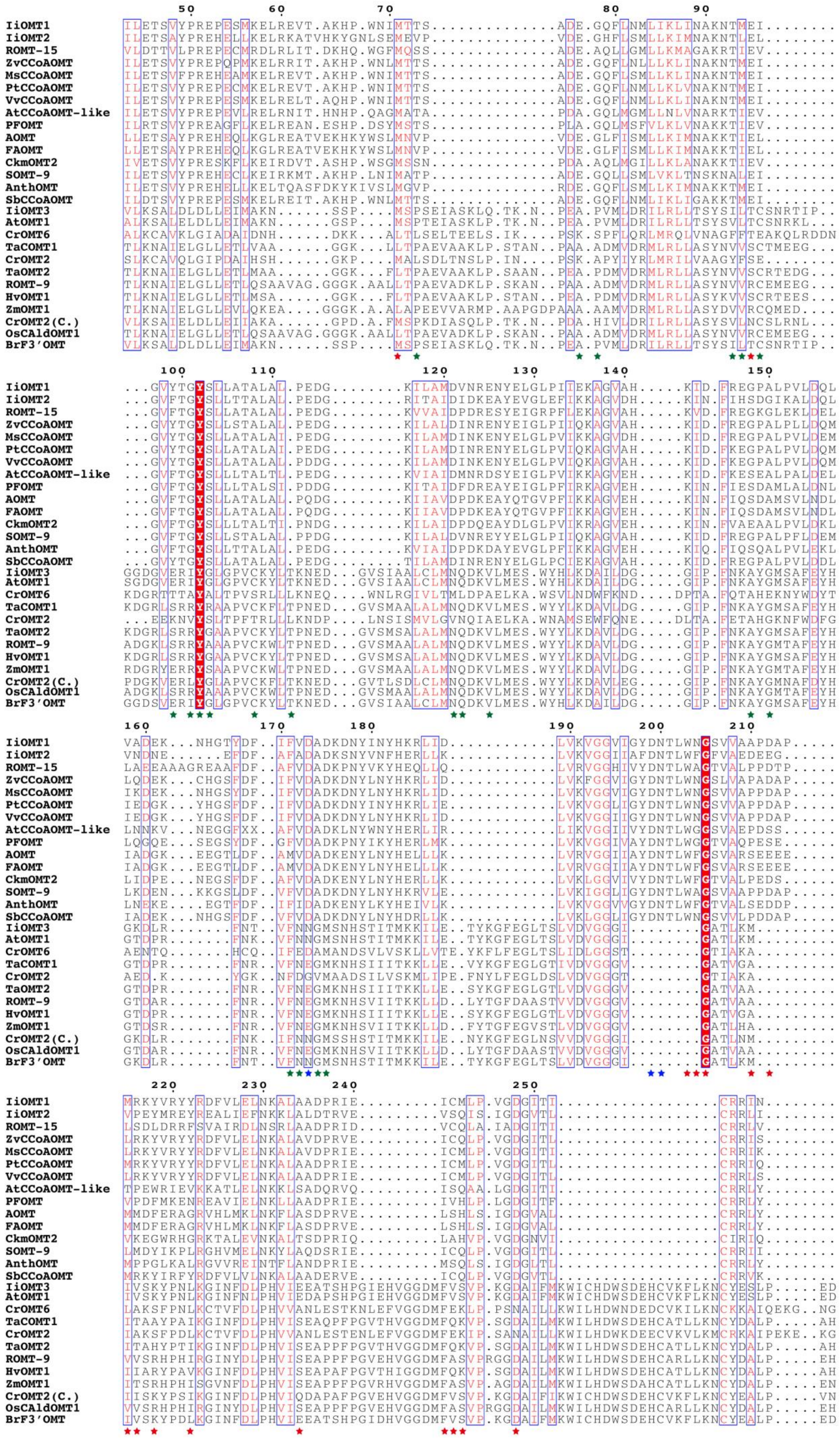

Figure S8. Sequence alignment of the IiOMTs with homologous proteins from other species in plants.

The substrate binding residues are marked by "★" below the sequence. A "★" indicates the SAM binding residues. A "★" indicates the metal binding residues.

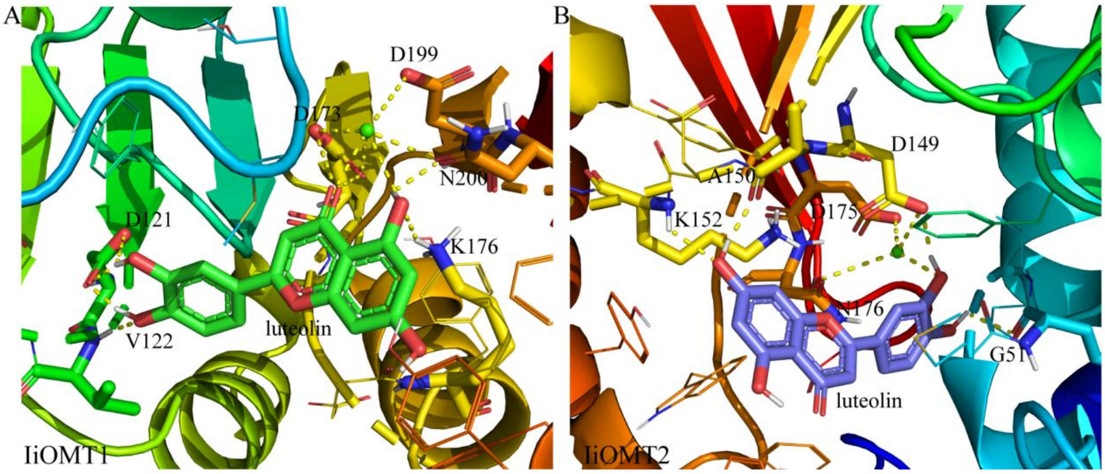

Figure S9. Molecular docking of IiOMT1 (A) and IiOMT2 (B) with luteolin as a ligand.

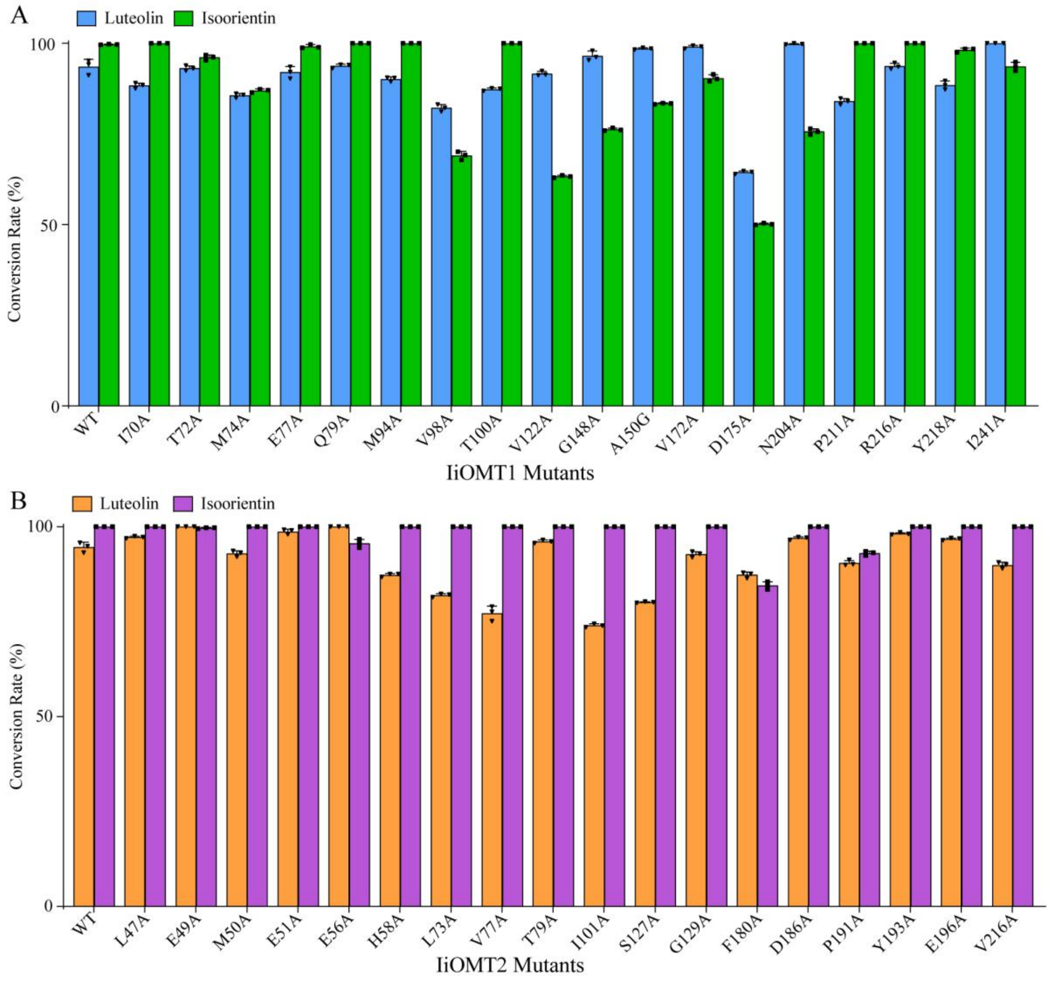

Figure S10. Site-directed mutagenesis to identification of the essential residues of IiOMT1 and IiOMT2.

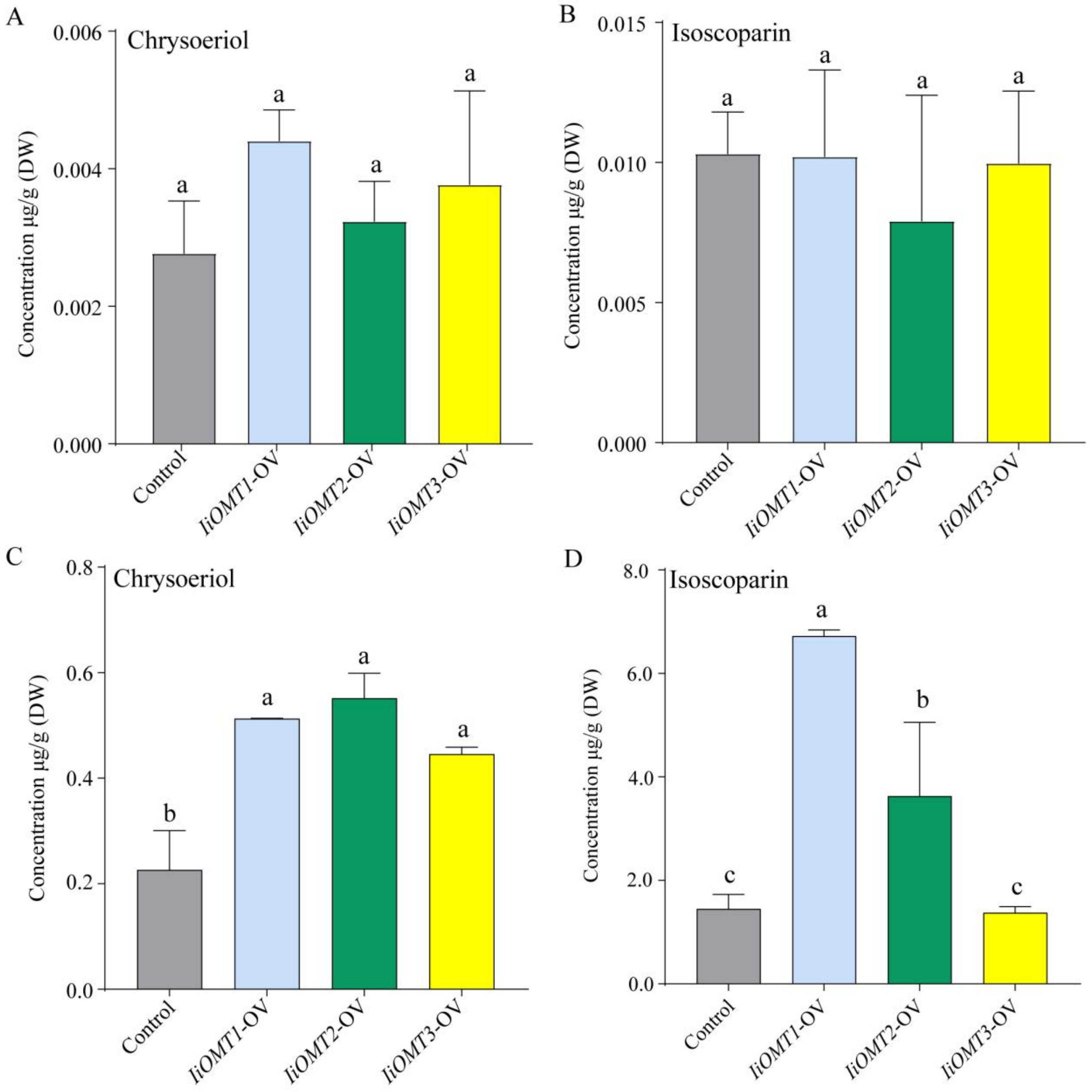

Figure S11. Transient overexpression analysis of *IiOMTs* without infiltrated substrates in *N. benthamiana*.

Effect of *IiOMT* gene transient overexpressing on the contents of chrysoeriol (A) and isoscoparin (B) without infiltrated substrates. Effect of *IiOMT* gene transient overexpressing on the contents of chrysoeriol (C) and isoscoparin (D) with infiltrated substrates. The data represent the mean ± standard deviation (SD) of three biological replicates. Different letters above the error bars indicate significant differences (p<0.05) according Tukey's test.

Table S1 List of primers used in this study.

Primers	Squences

Primers for cDNA amplification	
IiOMT1-F	caccgcgaacagattggaggtatggcgacgacgacaacag
IiOMT1-R	gctcgaattcggatcctctagtcaattgatccgacggcagat
IiOMT2-F	caccgcgaacagattggaggtatggccaatgaaataccttcca
IiOMT2-R	gctcgaattcggatcctctagttatataaggcgtctgcagagcgt
IiOMT3-F	caccgcgaacagattggaggtatgggatcaacggcggagac
IiOMT3-R	gctcgaattcggatcctctagtcagatcttcttgagcaactcaataac
Primers for Real-time qPCR	
IiOMT1-qF	attgggtttgccgatcatcg
IiOMT1-qR	tgtccttgtcagcatcaacg
IiOMT2-qF	ttgcgttcgacaacaccttg
IiOMT2-qR	ttcgactcgagtatccaaagcc
IiOMT3-qF	aagcctctcgaccaaacaag
IiOMT3-qR	acgttgatgcctttgaagcc
Primers for Site-directed mutagenesis PCR	
IiOMT1A150G-F	tttcagggaaggccctGGTcttcctgttc
IiOMT1A150G-R	Ccagggccttccctgaaatcgatcttg
IiOMT1D121A-F	caaaattctagccatgGCCgttaacagag
IiOMT1D121A-R	Gccatggctagaattttgccgtcttc
IiOMT1D173A-F	tgacttcatattcgttGCTgctgacaagg
IiOMT1D173A-R	Gcaacgaatatgaagtcatatgttcca
IiOMT1D199A-F	ggtgtgatcggctacGCCaacactctgt
IiOMT1D199A-R	Gcgtagccgatcacacctccaactttg
IiOMT1E77A-F	gacaacatcagcagatGCAgggcagtttc
IiOMT1E77A-R	Gcatctgctgatgttgtcattatgtt
IiOMT1G148A-F	gatcgatttcagggaaGCCcctgctcttc
IiOMT1G148A-R	Gcttccctgaaatcgatcttgtgagca
IiOMT1I241A-F	tgaccctcggatcgagGCAtgcatgctcc
IiOMT1I241A-R	GCctcgatccgagggtcagcagcgaga
IiOMT1K176A-F	attcgttgatgctgacGCGgacaactaca
IiOMT1K176A-R	GCgtcagcatcaacgaatatgaagtca
IiOMT1N200A-R	GCgtcgtagccgatcacacctccaact
IiOMT1N204A-F	cgacaacactctgtggGCCggttctgtcg
IiOMT1N204A-R	GCccacagagtgttgtcgtagccgatc
IiOMT1N200A-F	tgtgatcggctacgacGCCactctgtgga
IiOMT1R216A-F	tcctgatgcaccaatgGCGaagtacgttc
IiOMT1R216A-R	GCcattggtgcatcaggagcagcgacg
IiOMT1T72A-F	cccttggaacataatgGCAacatcagca
IiOMT1T72A-R	Ccattatgttccaagggtgttttgct

IiOMT1V98A-F	cacaatggagatcggcGCTtacactggct
IiOMT1V98A-R	Gcgccgatctccattgtgttcttggc
IiOMT1V122A-F	attctagccatggacGCTaacagagagaa
IiOMT1V122A-R	Gcgtccatggctagaattttgccgtct
IiOMT1V172A-F	atatgacttcatattcGCTgatgctgaca
IiOMT1V172A-R	Gcgaatatgaagtcatatgttccatg
IiOMT1Y99A-F	aatggagatcggcgttGCCactggctact
IiOMT1Y99A-R	GCaacgccgatctccattgtgttcttg
IiOMT2D100A-F	tcgtattactgcaataGCTattgacaaag
IiOMT2D100A-R	Gctattgcagtaatacgaccatcttca
IiOMT2D149A-F	tgatttcgcatttgcgGCTgcagacaagt
IiOMT2D149A-R	Gccgcaaatgcgaaatcaaactcctca
IiOMT2D175A-F	aggaatcattgcgttcGCCaacaccttgt
IiOMT2D175A-R	Gcgaacgcaatgattcctccaacctt
IiOMT2E49A-F	atatggcaacctaagcGCGatggaagttc
IiOMT2E49A-R	Gcgcttaggttgccatatttgtggac
IiOMT2E51A-F	caacctaagcgagatgGCAgttccagttg
IiOMT2E51A-R	Gccatctcgcttaggttgccatatttg
IiOMT2E56A-F	ggaagttccagttgatGCGggtcattttc
IiOMT2E56A-R	Gcatcaactggaacttccatctcgctt
IiOMT2F78A-F	tctcgagctcggtgttGCCaccggatact
IiOMT2F78A-R	GCaacaccgagctcgagagtgttctt
IiOMT2F180A-F	cgacaacaccttgtggGCTggttttgtgg
IiOMT2F180A-R	GCccacaaggtgttgtcgaacgcaatg
IiOMT2G129A-F	tttcatccattccgatGCTattaaggcct
IiOMT2G129A-R	Gcatcggaatggatgaaattgatctt
IiOMT2I101A-F	tattactgcaatagatGCTgacaaagaag
IiOMT2I101A-R	GCatctattgcagtaatacgaccatc
IiOMT2K152A-F	atttgcggatgcagacGCGtcaaactacg
IiOMT2K152A-R	GCgtctgcatccgcaaatgcgaaatca
IiOMT2N176A-F	aatcattgcgttcgacGCCaccttgtggt
IiOMT2N176A-R	GCgtcgaacgcaatgattcctccaac
IiOMT2P191A-F	ggacgaggaaggagttGCTgagtatatga
IiOMT2P191A-R	Caactccttcctcgtcctcagccaca
IiOMT2S127A-F	atcaatttcatccatGCCgatggtattaa
IiOMT2S127A-R	Catggatgaaattgatcttgtgatca
IiOMT2V77A-F	cactctcgagctcggtGCTttcaccggat
IiOMT2V77A-R	Gcaccgagctcgagagtgttcttagcg
IiOMT2V216A-F	ggatactcgagtcgaaGCCtctcagatct
IiOMT2V216A-R	Gcttcgactcgagtatccaaagccagt
IiOMT1D121N_F	gcaaaattctagccatgAACgttaacag
IiOMT1D121N_R	Tcatggctagaattttgccgtcttcggg
IiOMT1D173N_F	tgacttcatattcgttAATgctgacaagg

IiOMT1D173N_R	Taacgaatatgaagtcatatgttccatg
IiOMT1D199N_F	aggtgtgatcggctacAACaacactctg
IiOMT1D199N_R	Tgtagccgatcacacctccaactttgac
IiOMT1D248N_F	catgctccctgtgggtAATggaatcacta
IiOMT1D248N_R	Tacccacagggagcatgcatatctcga
IiOMT1N200D_F	tgtgatcggctacgacGACactctgtgg
IiOMT1N200D_R	Cgtcgtagccgatcacacctccaactt
IiOMT2D100N_F	tcgtattactgcaataAATattgacaaag
IiOMT2D100N_R	Ttattgcagtaatacgaccatcttcag
IiOMT2D149N_F	tgatttcgcatttgcgAATgcagacaagt
IiOMT2D149N_R	Tcgcaaatgcgaaatcaaactcctcat
IiOMT2D175N_F	aggaatcattgcgttcAACaacaccttg
IiOMT2D175N_R	Tgaacgcaatgattcctccaaccttcac
IiOMT2D233N_F	tcagatctccattggaAATggtgtcacg
IiOMT2D233N_R	Ttccaatggagatctgagagacttcga

Table S2. GenBank accession numbers of OMT proteins in Figure 2.

Genes	species	Accession number	Class type
HvF7OMT	*Hordeum vulgare*	CAA54616.1	I
AtOMT1	*Arabidopsis thaliana*	AAB96879.1	I
ChOMT	*Medicago sativa*	AAB48059.1	I
IOMT	*Medicago sativa*	AAC49927.1	I
CrOMT2	*Catharanthus roseus*	Q8GSN1	I
CrOMT6	*Catharanthus roseus*	Q6VCW3	I
ROMT-9	*Oryza sativa*	ABB90678.1	I
TaOMT2	*Triticum aestivum*	ABB03907.1	I
HvOMT1	*Hordeum vulgare*	ABQ58825.1	I
ZmOMT1	*Zea mays*	ABQ58826.1	I
TaCOMT1	*Triticum aestivum*	Q84N28.1	I
TaOMT2	*Triticum aestivum*	Q38J50.1	I

ShMOMT1	*Solanum habrochaites*	ADZ76433.1	I
ShMOMT2	*Solanum habrochaites*	ADZ76434.1	I
ObFOMT1	*Ocimum basilicum*	AFU50295.1	I
ObFOMT5	*Ocimum basilicum*	AFU50299.1	I
ObF8OMT-1	*Ocimum basilicum*	AGQ21572.1	I
ObFOMT3	*Ocimum basilicum*	AFU50297.1	I
ObFOMT4	*Ocimum basilicum*	AFU50298.1	I
CdFOMT5	*Citrus depressa*	BAU51794.1	I
PMT1	*Pinus sylvestris*	AQX17825.1	I
OsCAldOMT1	*Oryza sativa*	XP_015650053.1	I
CrOMT2	*Citrus reticulata*	ADK97702.1	I
PfOMT3	*Perilla frutescens*	QOE76460.1	I
GmIOMT1	*Glycine max*	NP_001353843.1	I
MsCCoAOMT	*Medicago sativa*	AAC28973.1	II
ZvCCoAOMT	*Zinnia violacea*	AAA59389.1	II
VvCCoAOMT	*Vitis vinifera*	CAA90969.1	II
PtCCoAOMT	*Populus tremuloides*	AAA80651.1	II
PFOMT	*Mesembryanthemum crystallinum*	AAN61072.1	II
SOMT-9	*Glycine max*	NP_001236240.1	II
AOMT	*Vitis vinifera*	ACO52469.1	II
FAOMT	*Vitis vinifera*	ADJ57332.1	II
CkmOMT2	*Cyclamen*	BAK74804.1	II
AtCCoAOMT-like	*Arabidopsis thaliana*	AAM64800.1	II
AnthOMT	*Solanum lycopersicon*	NP_001289828.1	II
PaMTH1	*Podospora anserina*	4QVK_A	II
SbCCoAOMT	*Sorghum bicolor*	5KVA_A	II
PaF6OMT	*Plagiochasma appendiculatum*	APX42106.1	II
MpOMT1	*Marchantia paleacea*	QCG81599.1	II
MeOMT1	*Marchantia emarginata*	QCG81600.1	II

HmOMT1	*Haplomitrium mnioides*	QCG81601.1	II
PaCCoAOMT1	*Polypodiodes amoena*	QCY65250.1	II
PaCCoAOMT2	*Polypodiodes amoena*	QCY65251.1	II
BrF3'OMT	*Brassica rapa*	XP_009132451	II
